# Industrial Robots and Regional Fertility in European Countries

**DOI:** 10.1007/s10680-023-09657-4

**Published:** 2023-03-28

**Authors:** Anna Matysiak, Daniela Bellani, Honorata Bogusz

**Affiliations:** 1 Interdisciplinary Centre for Labour Market and Family Dynamics, Faculty of Economic Sciences, University of Warsaw, Warsaw, Poland; 2 Department of Political and Social Sciences, University of Florence, Florence, Italy

**Keywords:** Fertility, Employment, Industrial robots, Technological change, Europe

## Abstract

In this study, we examine whether the long-term structural changes in the labour market, driven by automation, affect fertility. The adoption of industrial robots is used as a proxy for these changes. It has tripled since the mid-1990s in the EU, tremendously changing the conditions of participating in the labour market. On the one hand, new jobs are created, benefitting largely the highly skilled workers. On the other hand, the growing turnover in the labour market and changing content of jobs induce fears of job displacement and make workers continuously adjust to new requirements (reskill, upskill, increase work efforts). The consequences of these changes are particularly strong for the employment and earning prospects of low and middle-educated workers. Our focus is on six European countries: Czechia, France, Germany, Italy, Poland and the UK. We link regional data on fertility and employment structures by industry from Eurostat (NUTS-2) with data on robot adoption from the International Federation of Robotics. We estimate fixed effects linear models with instrumental variables in order to account for the external shocks which may affect fertility and robot adoption in parallel. Our findings suggest robots tend to exert a negative impact on fertility in highly industrialised regions, regions with relatively low educated populations and those which are technologically less advanced. At the same time, better educated and prospering regions may even experience fertility improvements as a result of technological change. The family and labour market institutions of the country may further moderate these effects.

**Supplementary Information:**

The online version contains supplementary material available at 10.1007/s10680-023-09657-4.

## Introduction

Over the last two decades, technological advancements in production, including cutting-edge industrial robots, have tremendously transformed the labour markets in advanced market economies, creating new career opportunities, but also inducing fears of job displacement (OECD, 2019). Only in the EU, the stock of industrial robots per 10.000 manufacturing workers has tripled since the mid-1990s reaching 114 in 2019 (International Federation of Robotics, 2020). Because of the scale and speed of automation and its possible consequences for workers, there has been an explosion of studies on how technological advancements in production affect employment (Acemoglu & Restrepo, 2020; Graetz & Michaels, 2018), wages (Dauth et al., 2021), social and economic inequalities (Aksoy et al., 2021; de Vries et al., 2020) and more recently workers’ physical and mental health (Abeliansky and Beulman, 2019; Gihleb et al., 2022). With this study, we contribute to this discussion by examining how automation, and more specifically the adoption of industrial robots, influences fertility, an outcome which so far has been largely neglected in the scientific debate.

In our view, automation may affect fertility since it alters the conditions of participating in the labour market and with it the economic well-being of the family and the strategies of its adult members adopted to combine paid work with care. Past research has clearly demonstrated that individuals tend to postpone or even abstain from having children during economic downturns (Cherlin et al., 2013; Sobotka et al., 2011), usually in response to an increase in unemployment and growing instability of employment (Adsera, 2004; Bellani, 2020; Matysiak et al., 2021; Schneider, 2015). The feeling of economic uncertainty may also hinder fertility decisions irrespective of the real economic conditions (Vignoli et al., 2020). Notably, fertility usually declines more strongly in response to worsening of employment prospects for men and young workers as well as in countries offering weaker social protection in case of a job loss (Alderotti et al., 2021; Comolli, 2017).

Past research has largely concentrated on examining fertility consequences of short-term changes in labour market conditions, caused by cyclical swings in the economy and reflected in upward and downward moves in (un)employment or work conditions. Much less has been done on how fertility reacts to long-term structural changes in the labour markets, driven, for instance, by globalisation or technological change. These changes may not necessarily affect (un)employment, but rather change the demand for workers’ skills. They may increase uncertainty, push workers into poorly paid low quality jobs or increase workers’ effort to catch up with quickly changing work guidelines and skill requirements (Autor et al., 2006; Green et al., 2022). In fact, Seltzer (2019) demonstrated that the cyclical approach performed very well in predicting a decline in fertility rates during the Great Recession in the USA, but completely failed in its aftermath when envisioning a fertility rebound.

This study contributes to the discussion on labour markets and fertility by investigating how the long-term structural changes in the labour market, driven by robot adoption, affect regional fertility. Robot adoption mirrors technological innovation and is a marker of economic and labour market transformation (Dottori, 2021). Following the International Federation of Robotics, we define industrial robots as fully autonomous machines that do not require a human operator (Jurkat et al., 2022). So far, little attention has been paid to this topic in fertility research. A notable exception among the published papers is the study by Anelli et al. (2021) who investigated the effects of the adoption of industrial robots on marriage and fertility in the USA. Our focus is on Europe, where, despite large cross-country diversity, workers are much better protected against job loss or poverty (Esping Andersen, 1990). By exploiting variation in robot penetration across NUTS-2 regions, we examine how robotisation influenced fertility in six European countries, namely Czechia, Germany, France, Italy, Poland and the UK. These countries differ in the penetration of automation, labour market and family policy regimes and gender norms. They also constitute good cases for examination as they provide a reasonable number of NUTS-2 regions for obtaining robust empirical findings (with Czechia pooled together with Poland).

## Literature Review

### Automation, Employment and Economic Uncertainty

The fear that automation will lead to a massive job destruction has been a concern for at least two centuries since the first industrial revolution began (OECD, 2019). Even though the industrial revolution didn’t, in the end, lead to unemployment, but to an expansion of job opportunities and improvement in living standards, fear of automation persisted. In the twenty-first century, we are facing a new wave of anxiety that robots will take over our jobs—this time it is about cutting-edge industrial robots (Dekker et al., 2017).

The adoption of robots and machines will indeed change the ways we work and change the demand for skills. Some jobs, in particular those which require performing routine tasks, will likely be destroyed or substantially changed (Acemoglu & Autor, 2011; Acemoglu & Restrepo, 2020). In the OECD countries, it was estimated that around 10–14% of jobs will be fully replaced by robots and for 25%—32% around 50–70% of tasks will be automated in the next two decades (Arntz et al., 2017; Nedelkoska & Quintini, 2018). Yet, automation does not only destroy jobs but also increases productivity and thereby facilitates job creation. The newly created jobs often require different skills, however. Most often there are non-routine highly cognitive skills which can be implemented in the expanding high tech sector, education or highly specialised customer service (Acemoglu & Autor, 2011). New jobs are also created in the lower-skill service sector (e.g. delivery workers, drivers), but they often offer poor social protection, are low paid and/or unstable (Autor, 2019).

Empirical research demonstrated the effects of automation on labour market outcomes to be unequivocal and clearly depend on workers’ education and skills, the sector they are employed in and the overall economic and institutional environment. Automation seems to exert particularly negative effects on employment and/or earning opportunities of low-and-middle educated workers, both in the USA (Acemoglu & Restrepo, 2020) and in Europe, though in the latter to a lower extent (Graetz & Michaels, 2018). Robots usually destroy jobs in manufacturing (Jung & Lim, 2020) but create new jobs in the service sector (for the US case see Acemoglu & Restrepo, 2020; for the UK see Kariel, 2021). As companies which adopt robots increase their productivity, they can invest more resources into product development, sales and marketing. Robots are thus indirectly increasing demand for workers who can fill in the jobs in highly specialised customer service and product development, not even mentioning the high tech workers who are able to design and operate industrial robots. Indeed, it was demonstrated that highly educated workers, performing nonroutine cognitive tasks, usually benefit from the ongoing changes (de Vries et al., 2020). Automation is also more likely to bring increases in employment in companies and regions which are more technologically advanced and better prepared to embrace the benefits brought about by technological progress. It was demonstrated, for instance, that regions with higher shares of knowledge and creative workers are better able to adapt to changes driven by digitalisation and thus are less vulnerable to automation shocks (Crowley et al., 2021). Last but not least, the effects of robotisation on employment and earnings may differ across countries and depend on their institutional settings. The labour substituting effect of robots tends to be stronger in countries with higher labour costs (Bachmann et al., 2022; Jung & Lim, 2020) and is argued to increase with a decline in employment protection legislation (Traverso et al., 2022).

Much less is known about how automation affects men’s versus women’s employment and earning opportunities, with few empirical findings suggesting mixed results. While Acemoglu and Restrepo (2020) find no gender differences in automation effects in the USA, Brussevich et al. (2019) argue that women in OECD countries may be more exposed to automation as they are more often employed in jobs which involve routine tasks (see also Piasna & Drahokoupil, 2017 for the same conclusions for the EU). Robotisation also seems to increase gender wage inequalities in Europe by disproportionately benefiting men in medium- and high-skill occupations (Aksoy et al., 2021). At the same time, however, there is evidence that young generations of women are moving away from the routine-intense jobs more quickly than men and take non-routine jobs in the service sector (Black & Spitz-Oener, 2010; Cortes et al., 2021) and that the pace of such job reallocation is faster in countries more advanced in robotisation (Aksoy et al., 2021).

Overall, whether the new wave of automation will indeed lead to declines in employment is not yet clear. There is evidence, however, that it increases turnover in the labour market, requires readjustment from workers and increases uncertainty. The aforementioned studies by Arntz et al (2017) and Nedelkoska and Quinitni (2018) demonstrate that robots substantially change the task content of jobs, modifying the demand for skills and requiring employees to acquire new qualifications and follow new guidelines. A study from Norway found that around 40% of workers fear being replaced by a machine, which lowers their job satisfaction (Schwabe & Castellacci, 2020). Abeliansky and Beulman (2019) demonstrated negative effects of robot adoption on workers’ mental health in Germany. Robot adoption was also found to increase death rates due to substance and alcohol abuse (Gihleb et al., 2022; O’Brien et al., 2022). Finally, the fear of robots was found to be particularly pronounced among the blue collar workers, most exposed to negative effects of automation, and in countries with weaker safety nets (Dekker et al., 2017).

### Automation and Fertility

A large body of the literature has provided evidence that weakening employment prospects, increase in unemployment and economic uncertainty lead to postponement of fertility or even lower fertility rates (Adsera, 2004; Comolli, 2017; Matysiak et al., 2021; Schneider, 2015). This is particularly true in countries offering weak safety nets for the unemployed (Mills et al., 2005). Growing instability of employment has also more negative consequences on fertility when it concerns men than women who, instead, may treat unemployment as an opportunity window for childbearing (Kreyenfeld & Andersson, 2014; Schmitt, 2012). These gender differences in the role of unemployment or precarious employment for fertility are, however, gradually in decline with an increase in women’s education, changing gender roles and growing instability of men’s employment (Oppenheimer, 1997). In a meta-study Alderotti et al. (2021) showed that in countries with high gender equality, such as Nordic Europe, or countries characterised by strongly unstable employment patterns among men, such as Southern Europe, women no longer use unemployment in order to have children. The same study showed that temporary contracts depress fertility more strongly if they are held by women than men.

Past research on labour market and fertility has, however, largely relied on such labour market indicators, such as (un)employment rate, wages or proportion of persons on specific contracts (e.g. temporary or part time). These indicators excel in identifying short-term cyclical economic conditions, but are less able to capture long-term structural changes in the labour markets, driven for instance by globalisation or technological change. These changes may not necessarily affect (un)employment, but may require workers to adjust to the changing demand for skills. They may thus increase uncertainty and workers’ effort to adapt new work guidelines and protocols or undertake training. New employment opportunities may open in front of some workers, while others may be pushed into poorly paid low quality jobs (Autor et al., 2006; Green et al., 2022). In particular, Seltzer (2019) showed that the cyclical approach performed very well in predicting a decline in fertility rates during the Great Recession in the USA, but failed when envisioning a fertility rebound in its aftermath. Instead, fertility continued to fall despite a steep decline in unemployment in the post-crisis period (until the breakdown of the Covid-19 pandemic). This phenomenon was apparently driven by long-term structural changes in the labour market, caused by globalisation and technological change. These changes started already before the Great Recession but accelerated throughout it as companies which implemented labour replacing technologies during the economic crisis were most likely to survive it (Hershbein & Kahn, 2018). With time, the displaced workers found employment in the lower-skill service sector, which resulted in a decline in unemployment, but these jobs were of lower quality, at least in the USA (Seltzer, 2019).

So far few studies have looked at how these long-term structural transformations in the labour market affect fertility. Among them the majority concentrated on changes caused by globalisation, in particular the detrimental role of import competition with China for employment opportunities of middle-skilled workers, mostly male, in goods-producing industries. Studies consistently showed that increased import competition led to a decline in fertility, largely by a declining marriage value of men (Autor et al., 2019; Giuntella et al., 2022; Piriu, 2022). Researchers’ interest in how technology-driven labour market changes affect fertility has been even scarcer. On one hand, it has been shown that technological complexity, that reflects the capacity to innovate, develop and create job opportunities, is positively associated with fertility (Innocenti et al., 2021). This is because it fosters a fertility-friendly context characterised by better employment prospects. On the other hand, however, technological upgrading driven by automation is likely to increase turnover in the labour market, increase uncertainty and force workers to re-skill, which, in turn, may decrease fertility. In the only published empirical study on the effect of robotisation on fertility, Anelli et al. (2021) demonstrate that an increase in the adoption of industrial robots in the USA led to an increase in cohabitation and divorce and a decline—though not significant—in the number of marriages. Their findings also point to a decline in marital fertility and an increase in out-of-wedlock births.

## Country Context

Our study is situated in six European countries, namely Czechia, Germany, France, Italy, Poland and the UK. This country choice is driven by the desire to cover European countries which represent different labour market and family policy regimes and which also differ in the advancement of robot adoption. At the same time, we faced data restrictions. Conducting a regional level analysis, we were restricted to the choice of only bigger European countries with a large number of NUTS-2 regions. Furthermore, due to the choice of the IV strategy (for details, see Sect. 5.2) we were not able to pool European countries into groups (except for Czechia and Poland).

Among the selected countries France and UK have had the highest fertility for about four decades (with TFR oscillating between 1.7 and 2.0), though on a slow but gradual decline since the onset of the Great Recession. Germany and Italy had been the lowest low fertility countries (with TFR below 1.35) since the mid-1980s and Czechia and Poland since the late 1990s/early 2000s. However, while Germany and in particular Czechia experienced some increase in fertility over the last 15 years, Italy and Poland remained at the fairly low levels with TFR oscillating between 1.25 and 1.45 (Eurostat, 2022).

The analysed countries also represent different welfare regimes which define the extent to which workers are protected against a job loss and supported in case of unemployment, all of which may matter for their fertility decisions (Adsera, 2005; Bastianelli et al., 2022). Germany and France are typically classified into the conservative/employment-centred regimes (Amable, 2003; Esping-Andersen, 1990; Walther, 2006), based on strong employment protection and coordinated bargaining systems which allow for a “solidaristic wage setting” (Amable, 2003: 15). The two countries tend to offer generous income support for the unemployed and institutional support in job search (Tamesberger, 2017). Employment protection is also high in Italy, but is strictly directed at protecting workers on permanent contracts, leaving workers on temporary contracts often trapped in the secondary labour market (Pinelli et al., 2017). The UK, instead, is an example of liberal welfare state (Esping-Andersen, 1990), with a very low employment protection and low public support for the unemployed, offered only to those in the highest need (Caroleo & Pastore, 2007). Finally, Czechia and Poland belong to the post-socialist transitional regime with strong market orientation, low levels of state intervention, weak unions and limited support for the unemployed (Visser, 2011), providing rather low support for the unemployed (Tamesberger, 2017). They also display much lower labour costs than the remaining countries (Eurostat, 2022).

Family policies and the gender norms represent another element of the country context which may affect fertility responses to the changing labour market conditions. Whereas France stands out for its very good childcare coverage, Germany for a long time adhered to a modernised male breadwinner policy and only recently started to invest in childcare (Fagnani, 2012). Consequently, while it is common for mothers in France to work full time, many women in Germany switch to part-time jobs after they become mothers (Fagnani, 2007). In Italy, childcare is seen as a private issue, which results in strong gender inequalities both in paid and unpaid work (Menniti et al., 2015). Childcare provision in the UK is also weak and care usually has to be purchased on the market (Yerkes & Javornik, 2019). Mothers usually work part-time or make use of flexible work arrangements which are available in the UK on a wider scale than in other studied countries (Chung & Horst, 2018). Poland and Czechia also display low childcare provision (Szelewa & Polakowski, 2008). Interestingly, mothers usually return to full-time employment after birth though in Czechia much later than in Poland (Matysiak, 2011).

**Fig. 1:**
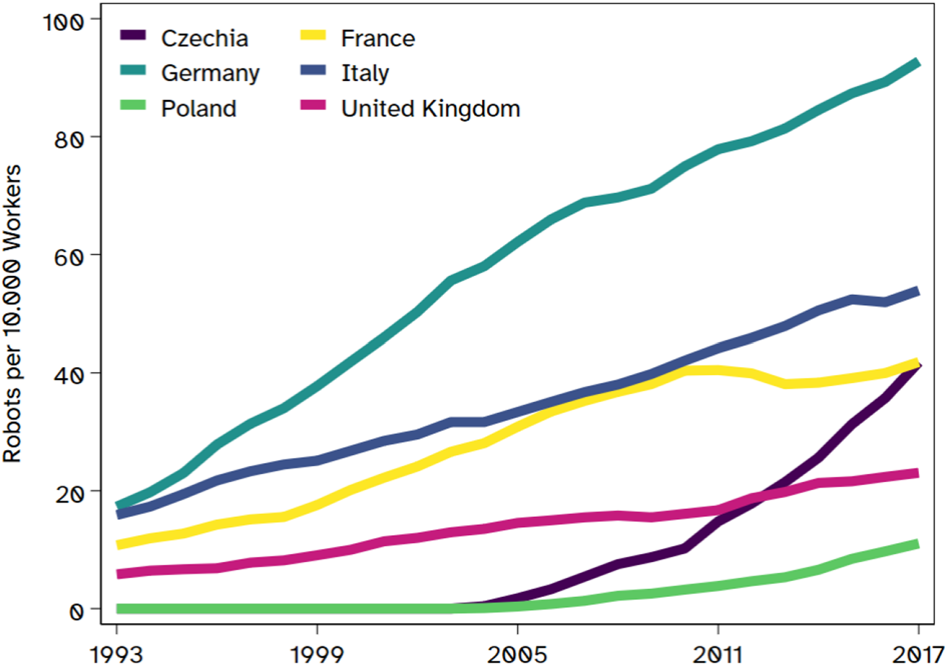
Industrial robot penetration in 6 European countries by calendar year. *Sources: International Federation of Robotics (IFR) and Eurostat. Calculated by summing robot stocks and employment for the following 1 digit industries: manufacturing, mining and quarrying, electricity, gas, water supply, and construction. Time series are constrained by data availability, as IFR publishes robot stock from 1993 onwards. Figure prepared by the authors in Stata*

**Fig. 2:**
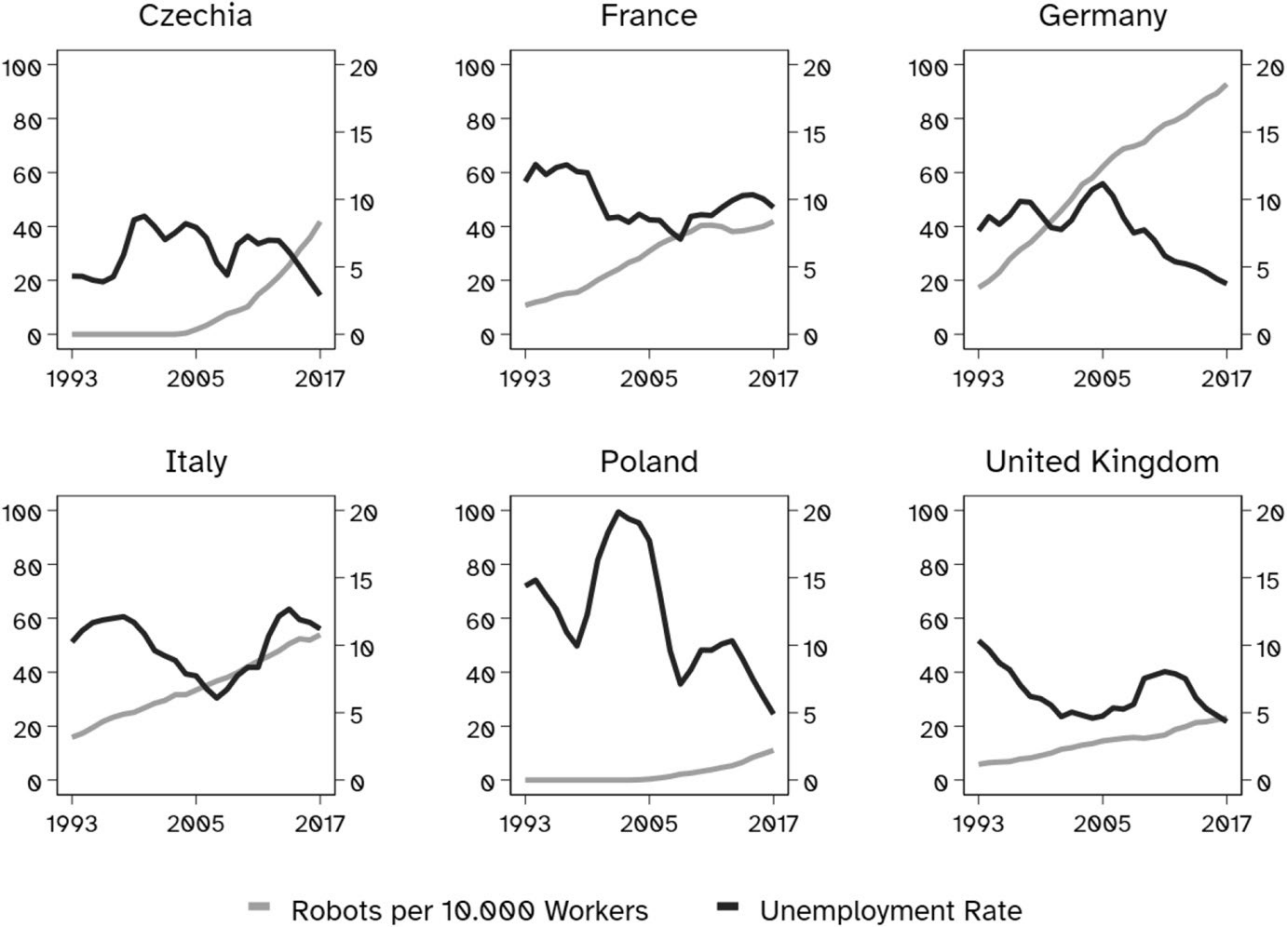
Robot penetration (left y axis) vs unemployment (right y axis) by country in time. Note: Robot stocks are summed up for the following 1 digit industries: Manufacturing, Mining and quarrying, Electricity, gas, water supply, and Construction. Source: International Federation of Robotics and Eurostat. Figure prepared by authors in Stata

Finally, the analysed countries differ in the robot penetration. The process of robot adoption in the old EU member states (Germany, France and Italy) and the UK started in the early 1990s (see Fig. 1). In all these countries, robots are predominantly employed in the automotive industry, apart from Italy where the allocation of robots across industries is more balanced with 26% in the metal, 17% in the automotive and 12% in the plastic and chemical industry (International Federation of Robotics, 2020). Germany is a clear leader in robot adoption worldwide (Dauth et al., 2021). It is followed by France and Italy where the robot penetration, measured by the number of robots per 10,000 employees, in 2019 was around half of that in Germany. Even lower penetration is observed in the UK which is an example of the Western European country with relatively slow adoption of industrial robots. The two post-socialist countries, Czechia and Poland, also display lower levels of robotisation, but the process of robot adoption started much later there, in the late 2000s. Robotisation in Czechia was very dynamic, due to the rapid development of its automotive industry, with the penetration rate surpassing the French one in 2017. The process in Poland was slower though gradual. Interestingly, in none of the studied countries did an increase in robot adoption go hand in hand with an increase in unemployment (see Fig. 2). Neither did robot penetration change during the Great Recession. Instead, we observed a gradual increase in robot adoption in all analysed countries alongside cyclical movements in unemployment. This observation confirms that robotisation does not necessarily reflect the same phenomenon as unemployment.

## Research Objectives and Hypotheses

In this study, we extend the work by Anelli et al. (2021) and examine the effects of long-term structural changes in the labour market, driven by adoption of industrial robots, on regional fertility rates in six European countries—Czechia, France, Germany, Italy, Poland and the UK. As we demonstrated in Sect. 2.1, automation may benefit certain groups of workers (e.g. highly educated, working in the service sector) and diminish the earning/employment opportunities of the others (e.g. low and middle educated workers in the manufacturing sector). We thus do not expect it to affect regional fertility rates in any uniform way. Instead, we anticipate the fertility effects of robot adoption to depend on the structural conditions of the regional labour markets. First, we expect robot adoption to exert more negative/less positive effects on fertility in those regions which used to have large employment in manufacturing before the onset of robotisation **(H1).** This expectation is formed due to the fact that industrial robots are largely employed in manufacturing, leading to a larger job destruction, turnover and uncertainty there rather than in the service sector. Second, we hypothesise that the negative (positive) fertility effects of robot adoption will be more (less) evident in regions where the proportion of men employed in manufacturing at early stages of automation was larger, making men more exposed to robotisation **(H2)**. This is because fertility is less likely to decline in a reaction to a deterioration in women’s than men’s employment conditions. Next, we expect stronger fertility declines/weaker fertility increases in response to robot adoption in regions with a larger proportion of low and middle educated workers **(H3)** since they are the ones which are mainly negatively affected by automation, either by being at risk of job displacement or having to compete with displaced workers for jobs. Last but not least, we anticipate that fertility effects of robot adoption depend on the region’s capacity to embrace technological change. Consistently with past research showing that employment effects of robot adoption are weaker or even positive in regions which invest in modern technologies, we expect that fertility will be less likely to decline/more likely to increase in response to automation in technology- and knowledge-intensive regions **(H4)**. Finally, fertility effects of robot adoption may also vary across the studied countries since they display substantial differences in welfare regimes, the gender normative context and penetration of automation. We abstain, however, from formulating specific hypotheses on the role of the specific cross-country differences for our findings since a comparison of only six countries which vary in numerous important dimensions precludes testing such hypotheses. We rather discuss our findings from the perspective of the cross-country differences presented in Sect. 3.

## Methodology

### Data

Our study is based on regional NUTS-2 data. The nomenclature of territorial units for statistics (NUTS) is a hierarchical system for dividing up the economic territory of the European Economic Area, the UK, and Switzerland for the purpose of data collection and socio-economic analyses. NUTS-2 regions are roughly equally populated, with population ranging from 0.8—3 million, and these are the smallest geographical units for which employment data are available in Eurostat for all 6 countries of our interest. We observe the countries fairly since the start of the robotisation till 2017. This means we cover the years 1997–2017 for the old EU member states and the UK and 2007–2017 for Czechia and Poland. Covering fully the 1990s for the old EU member states was not possible due to data availability.

To measure fertility, we use TFR and the age-specific fertility rates for the following age groups: 20–24, 25–29, 30–34, 35–39, 40–44, 45 + . These data have been provided by Eurostat at the NUTS-2 level since 1990. They are computed by combining national statistics on births by mother’s age and population of women by age. They are fairly complete with some missing data in fertility of women aged 45 + (around 10% of all observations). We use simple linear interpolation to supply them.

To measure worker’s exposure to automation we use data on industrial robot stocks provided by the International Federation of Robotics (henceforth: IFR). Industrial robots are defined by IFR as fully autonomous machines that do not require a human operator. Their main tasks are handling operations and machine tending (55% of all European robots fall into this category) and welding and soldering (22% of all European robots) (Jurkat et al., 2022). IFR provides annual data on the operational stock of industrial robots by country and industry since 1993. The industries are coded according to the International Standard Industrial Classification of all economic activities (ISIC, UN, 2008). The stocks of robots are provided by IFR at 1 digit level for all ISIC industries, and max 3 digits for manufacturing industries. The IFR data is complete. We utilise records at 1 digit for three following ‘heavy’ industries: mining and quarrying, electricity, gas, water supply, and construction. We utilise records at 2 digits for the remaining 13 manufacturing industries^1^ to match our regional employment structure data, which is also coded in 2-digit industry categories. We don’t include non-industrial categories such as Services, Public Administration, or Education, as those industries employ predominantly service, not manufacturing robots, and at a much smaller scale than robots operating in manufacturing or ‘heavy’ industries (Hajduk and Koukolova, 2015).

The data on robots are linked to data on regional employment structures by industry using the methodology developed by Acemoglu and Restrepo (2020) and described in detail in Sect. 5.2. Eurostat has provided NUTS-2 regional employment structures by 2-digit industry codes classified according to Nomenclature of Economic Activities (NACE Rev. 1.2 before 2008, NACE Rev. 2 after 2008) since 1986. We reclassify these data to the ISIC classification to match them to robot stocks. Moreover, since our main covariate (explained in detail in Sect. 5.2) relies on summation of employment numbers over time, we impute missing records of the regional employment structure. Finally, changes in the past NUTS classifications require reclassifying regional codes to one, consistent version. Both reclassifications and the imputation are described in detail in the Appendix in Tables 6 and 7.

Besides fertility rates, Eurostat online database provides us also with NUTS-2 level controls by calendar year, as well as potential moderators, which we interact with our main explanatory variable in order to test our research hypotheses. We include the following set of controls at the regional level: share of population aged 15–24, share of population aged 25–49, share of population aged 50 + , share of highly educated (ISCED levels 5–8), ratio of share of highly educated women to share of highly educated men, the square of the latter and women’s economic activity rate. The variables denoting population structure by age are introduced to control for any variation in population exposed to childbearing. We also account for the population education level given the educational gradient in fertility (Wood et al., 2014). The share of highly educated women relative to highly educated men and the square of this ratio aim at capturing the difficulties to find a partner in regions with better educated female population (Bellani et al., 2017) given that partners tend to form unions if they have similar education levels (de Hauw et al., 2017). Finally, women’s economic activity rate is also tightly linked to fertility.

The potential moderating variables are settled at the regional level as well. They are the initial (measured around the onset of robot adoption) proportion of workers employed outside of manufacturing (used to test H1), the initial proportion of women employed in manufacturing over the proportion of men in manufacturing (H2), proportion of highly educated persons (time-varying) (H3) and the proportion of workers employed in technology- and knowledge-intensive sectors (time-varying) (H4). The control and moderating variables are fairly complete. Any missing values were imputed via linear interpolation. This was done in 14% of cases for population structure by education, and max. 25% for employment data. There are no cases when the entire time series for specific regions are missing.

After accounting for the NUTS reclassifications and excluding foreign territories (see Table 7 in the Appendix), we have data for 34 NUTS 2 regions in Germany, 22 in France, 20 in Italy, 35 in the UK, 16 in Poland, and 8 in Czechia. We pool the data for Czechia and Poland due to the smaller number of regions in the two post-socialist countries and their similarities when it comes to labour market and family policy institutions, economic developments and delayed start of automation in comparison with Western Europe. In total, we have 680 observations for Germany, 440 for France, 400 for Italy, 700 for the UK and 240 for Czechia and Poland jointly.

### Methods

Our methodology relies on regressing fertility rates against workers’ exposure to robotisation as well as a set of control variables mentioned in Sect. 5.1, separately for Germany, Italy, France, the UK and the group formed by Czechia and Poland.

We quantify workers’ exposure to robotisation following the methodology developed by Acemoglu and Restrepo (2020) and used, among others, in Dauth et al. (2021), Anelli et al. (2021), and O’Brien et al. (2022):

1 $$Exposure\,to\, robots_{r,t} = \mathop \sum \limits_{i = 1}^{N} \frac{{empl_{{r,i,t_{0} }} }}{{empl_{{r,t_{0} }} }}\left( {\frac{{robots_{i,t}^{C} }}{{empl_{{i,t_{0} }} }}} \right)$$

where $robots_{i,t}^{C}$ is the country-level stock of robots across industries in year $t$; $empl_{{i,t_{0} }}$ identifies the total number of workers (in 10 thousands) employed in sector $i$ in $t_{0}$, i.e. at the start of the robotisation (hereafter initial) and $\frac{{empl_{{r,i,t_{0} }} }}{{empl_{{r,t_{0} }} }}$ denotes the initial distribution of employment in industry *i* across regions. Effectively, $\frac{{robots_{i,t}^{C} }}{{empl_{{i,t_{0} }} }}$ captures robots adopted in industry *i* and country *c* replacing its initial employment, while $\frac{{empl_{{r,i,t_{0} }} }}{{empl_{{r,t_{0} }} }}$ disaggregates it onto regions. We set $t_{0}$ to 1994 for Western European countries and to 2004 for Czechia and Poland, as those are years when robotisation started in those respective countries (see Sect. 3). The measure defined in *Eq. **1* is known as “shift-share instrument” or “Bartik instrument” (Goldsmith-Pinkham et al., 2020).

While exposure to robots is already considered exogenous, as its variation relies on employment shares before robotisation had started, concerns about endogeneity of $robots_{i,t}^{C}$ might still appear, i.e. when external factors affect both the robot adoption and fertility. These may be domestic or sector-specific shocks, such as policy changes. To address this issue, we follow Acemoglu and Restrepo (2020) and instrument the industry-specific stock of robots in country c $robots_{i,t}^{C}$ with industry-specific stock of robots in other countries, which serve as a proxy for advancements in robotisation in developed economies.Choosing the right country for instrumenting robot adoption in Western European countries turned out to be challenging, however. The US’ industry-specific stocks of robots could not be used for this purpose since robots (relative to workforce) in that country were used on a smaller scale than in Western Europe (International Federation of Robotics, 2020)—thus the USA cannot be considered as a pioneer of robotisation which the Western European countries would follow. Some of the East Asian economies are more advanced in robotisation than Western Europe (e.g. South Korea), but they adopt robots in other industries than European countries. We are thus uncertain about whether Europe will follow their path. We adopt the strategy suggested by Dauth et al. (2021) who used industry-specific stocks of robots from several advanced economies as instruments of robot stocks in Germany (overidentified IV model). We thus build an overidentified model for each country with $k =${Germany, France, UK, Italy, Spain, Sweden, Norway, Finland, United States of America} instruments. In models for Germany, France, UK, and Italy, we exclude the country of interest and the USA, and thus apply 7 instruments. In models for Poland and Czechia, all 9 instruments are applied. Those external instruments are likely relevant, as industrial robots are manufactured by only a few international companies, which set global trends in industrial robot adoption. Thus, robot adoption in one developed economy is a good proxy for robot adoption in another one, with a similar socio-economic context. The proposed set of instruments should also be valid, as there is no reason to expect that robot adoption in one developed economy has a direct influence on fertility rates in another one. To test the instruments’ relevance and validity of the overidentifying restrictions, we compute Kleibergen-Paap rk Wald F statistic, and Hansen J statistic (Kleibergen & Paap, 2006; Sargan, 1958; Wooldridge, 2010) and report it along with full model results in the Appendix (Tables 8, 9, 10, 11, 12). Even though this strategy for instrumenting our variable of interest resulted in relevant and valid instruments, it also has a drawback. Namely, we were not able to pool all European countries and estimate one model as that would leave us with collinear sets of instruments, which would be endogeneous and thus of little use.

Our model takes the following form:

2 $$fertility_{r,t} = \alpha Exposure\,to\,robots_{r,t - 2} + \beta Controls_{r,t - 1} + \eta_{r} + v_{t} + \varepsilon_{r,t}$$

where $fertility_{r,t}$ denotes regional total and age-specific fertility rates, $\alpha$ is our parameter of interest capturing the effect of workers’ exposure to robotisation on fertility in region *r*, $\eta_{r}$ corresponds to region individual effects and $v_{t}$ are time dummies. In order to test hypotheses H1-H4, we interact $Exposure to robots_{r,t - 2}$ with the potential moderators listed in Sect. 5.1. In all models, we control for a set of demographic and socioeconomic characteristics of a region, $Controls_{r,t - 1}$, enumerated in Sect. 5.1, which may confound the effects of robot penetration on fertility. They are lagged by 1 year to avoid simultaneity issues. At the same time, we lag the exposure to robots by 2 years to account for the pregnancy and the fact that, once exposed to labour market changes, workers might take some time to decide whether to have a child or not. *Equation **2* is estimated using the two-stage least squares approach with a fixed effects “within” estimator (Wooldridge, 2010). Standard errors are clustered at the region level to acknowledge for within-region dependence of the observations and robustify the model to serial correlation.

## Results

Our full model estimates along with the IV tests are displayed in Tables 8, 9, 10, 11, 12 in the Appendix (basic models as expressed by Eq. 2) and Tables 1–20 in the Online Supplementary Material (models with interactions). In all 175 regressions for the different countries and fertility rates, the instrument was relevant (as indicated by the Kleibergen-Paap rk Wald F statistic) and the overidentifying restrictions were valid with the Hansen J p-value exceeding the 5% significance level in 153 regressions, and the 1% in 8 cases. In 14 cases, it was not possible to conduct the Hansen J test, due to the fact that the number of clusters (regions) was smaller than the sum of the number of exogenous regressors and the number of excluded instruments (Baum et al., 2002; Frisch & Waugh, 1933). Those 14 cases correspond to the models for Italy and Czechia with Poland in which we introduced two interactions at once to test the H2. However, given that the overidentifying restrictions were valid in all other cases for those country samples, it is reasonable to assume that they are valid also in the remaining 14 cases.

### Overall Effects of Robot Adoption on Fertility

We find few rather small effects of robot adoption on fertility (Table 1). Total fertility is affected significantly only in Italy. This effect is negative: An increase in workers' exposure to robots by 1 robot per 10.000 workers reduces the total fertility rate by 0.00118. This effect is entirely driven by the negative effect of automation on fertility at young ages, in particular in the 25–29 group. Apart from Italy, we also find negative fertility effects in Germany, the leader of robot adoption worldwide, for certain age-specific fertility rates. These effects are weaker and, in contrast to Italy, emerge only at older ages (i.e. for age groups 35–39 and 40–44). We do not find significant negative effects on fertility in other countries of our interest. In some of them, we even identify a significant positive influence of robots on fertility at higher ages. For instance, an increase in exposure to robots by 1 robot per 10.000 workers results in an increase in 35–39 fertility rate 0.00025 in Czechia and Poland and a gain in the 40–44 fertility rate by 0.00039 in the UK. We don’t observe any statistically significant findings for France.

**Table 1:** Exposure to robots ($\alpha$) coefficients from basic 2SLS models (Eq. 2)

Country	TFR	FR 20–24	FR 25–29	FR 30–34	FR 35–39	FR 40–44	FR 45 +
Germany	− 0.00016	0.00004	0.00002	− 0.00002	− 0.00011***	− 0.00005***	− 0.000001
France	0.00003	− 0.00010	0.00009	0.00012	− 0.00001	− 0.00004	0.000003
Italy	− 0.00118*	− 0.00020	− 0.00090***	− 0.00012	0.00014	− 0.00005	0.00001
UK	0.00168	− 0.00087	0.00079	0.00133	0.00109	0.00039*	− 0.000002
Czechia & Poland	0.00053	0.00010	− 0.00044	0.00050	0.00025*	− 0.00005	− 0.00001

***1% **5% *10%. Sample sizes: 680 observations for Germany, 440 for France, 400 for Italy, 700 for the UK, and 240 for Poland and Czechia jointly.

### Workforce Sectoral Composition

Since robots are mostly employed in manufacturing, we hypothesised that the negative fertility effects will be most likely to emerge in regions with large manufacturing sectors (H1). The respective findings are presented in Table 2. The coefficients in rows entitled 'Exposure to robots' show the main fertility effects of robotization in regions with high initial employment in manufacturing and the interaction term beneath informs us about the extent to which the effect of robotization differs from the main effect in regions where the initial proportion of persons employed in manufacturing in the region was 1 pp lower.

With few exceptions, our findings are largely consistent with our hypothesis H1.

**Table 2:** Exposure to robots ($\alpha$) and its interaction with the initial (start of observation period) share of workers employed in manufacturing

Country	Measure	TFR	FR 20–24	FR 25–29	FR 30–34	FR 35–39	FR 40–44	FR 45 +
Germany	Exposure to robots	− 0.0022*	− 0.0012***	− 0.00137***	0.00037	0.00009	− 0.000029	− 0.00000
	Exposure to robots # Initial share of workers out of manufacturing	0.00003**	0.00002***	0.00002***	− 0.00001	− 0.00000	− 0.00000	0.00000
France	Exposure to robots	0.00163	0.00062	0.00212	0.00013	− 0.00082	− 0.00045**	− 0.00008*
	Exposure to robots # Initial share of workers out of manufacturing	− 0.00002	− 0.00001	− 0.00003	− 0.00000	0.00001	0.000006*	0.000001*
Italy	Exposure to robots	− 0.00264	− 0.00051	− 0.00201	− 0.00085	0.00069	0.00039**	− 0.00013**
	Exposure to robots # Initial share of workers out of manufacturing	0.00002	0.000005	0.00002	0.00001	− 0.000007	− 0.00001**	0.000002***
UK	Exposure to robots	− 0.0223**	− 0.00584	− 0.00094	− 0.00088	− 0.00384	− 0.00155	0.00012
	Exposure to robots # Initial share of workers out of manufacturing	0.00031**	0.000065	0.00002	0.00003	0.00006	0.00003	− 0.00000
Czechia & Poland	Exposure to robots	0.00627	0.00295***	− 0.00337**	0.00275	0.00251***	0.00004	0.00001
	Exposure to robots # Initial share of workers out of manufacturing	− 0.00009*	− 0.00005***	0.00005**	− 0.00004	− 0.00004***	− 0.00000	− 0.00000

***1% **5% *10%. Sample sizes: 680 observations for Germany, 440 for France, 400 for Italy, 700 for the UK, and 240 for Poland and Czechia jointly.

We observe a clearly negative effect of robot adoption on total fertility in those German regions which were initially highly industrialised. It is strongly driven by fertility reduction at young ages (20–24 and 25–29). This negative effect is significantly weaker in regions with a smaller initial proportion of workers employed in manufacturing. We also detect some negative fertility effects of robots in the French and British regions with initially large manufacturing sectors. In the UK, the negative effects on age-specific fertility in those regions are not significant but the negative effect on total fertility is significant. In France, they emerge at the highest reproductive ages: 40–44 and 45 + . In Italy, most of the effects in highly industrialised regions are insignificant except for those at higher reproductive ages where the pattern is unclear (positive effect of robot adoption in highly industrialised regions at ages 40–44 and negative at ages 45 +). Some inconsistency is also detected in Czechia and Poland though it seems that the effects of robot adoption there tend to be rather positive in highly industrialised regions: The main effects at all reproductive ages, but for 25–29, are positive though significant only at ages 20–24 and 35–39.

### Gender Composition of Manufacturing Workers

Next, we expected that fertility effects of robot adoption will be more negative in regions where men were more exposed to automation than women (H2). The findings which allow to verify this hypothesis are presented in Table 3. The coefficients in rows entitled "Exposure to robots' display fertility effects of robotisations in regions with high initial employment in manufacturing where in addition employment in manufacturing was dominated by men. The following interaction terms inform us to what extent the effect of robotization differs from the main effect in regions where the initial proportion of persons employed in manufacturing in the region was 1 pp lower/initial ratio of women over men employed in manufacturing was by 1 pp. higher.

 Apart from the UK and the cluster built by Czechia and Poland, we do not find evidence for hypothesis H2. Our findings even suggest the reverse, namely that robot adoption in Germany, France and Italy leads to stronger fertility decline in regions where the initial ratio of women’s to men’s employment share in manufacturing was larger. These negative effects, obtained net of the regional employment in manufacturing and women’s activity rate, are largely significant at young reproductive ages. Interestingly, in Italy and to some extent in France we even find traces of positive effects of robot adoption in regions with initially large manufacturing sectors which are dominated by men.

**Table 3:** Exposure to robots ($\alpha$), interaction of exposure to robots with the initial share of workers employed out of manufacturing and interaction of exposure to robots with the initial ratio of women’s to men’s employment share in manufacturing

Country	Measure	TFR	FR 20–24	FR 25–29	FR 30–34	FR 35–39	FR 40–44	FR 45 +
Germany	Exposure to robots	0.00079	− 0.00041	− 0.00018	0.00064	0.00053	0.00000	0.00001
	Exposure to robots # Initial share of workers out of manufacturing	0.00001	0.00001**	0.00001**	− 0.00001	− 0.00001	− 0.00000	− 0.00000
	Exposure to robots # Initial ratio of women's versus men's share in manufacturing	− 0.0035***	− 0.00093*	− 0.00141***	− 0.00031	− 0.00052**	− 0.00004	− 0.00001
France	Exposure to robots	0.0049	0.00188*	0.00352**	0.0008	− 0.00098*	− 0.00056**	− 0.00012**
	Exposure to robots # Initial share of workers out of manufacturing	− 0.00002	− 0.00000	− 0.00003	− 0.00000	0.00001	0.000005*	0.000001*
	Exposure to robots # Initial ratio of women's versus men's share in manufacturing	− 0.00681**	− 0.00307***	− 0.00292*	− 0.00122	0.00049	0.00036	0.00011**
Italy	Exposure to robots	0.0144***	0.00535***	0.0067***	− 0.00056	0.00038	0.00116***	− 0.00014*
	Exposure to robots # Initial share of workers out of manufacturing	− 0.00011**	− 0.00004**	− 0.00005***	0.00001	− 0.00000	− 0.00001***	0.000002**
	Exposure to robots # Initial ratio of women's versus men's share in manufacturing	− 0.0137***	− 0.00462***	− 0.00693***	− 0.00039	0.00025	− 0.0006***	0.00002
UK	Exposure to robots	− 0.0378***	− 0.0103	− 0.00187	− 0.00319	− 0.00486	− 0.00202	− 0.00024
	Exposure to robots # Initial share of workers out of manufacturing	0.00042***	0.0001	0.00003	0.00004	0.000069	0.000028	0.000001
	Exposure to robots # Initial ratio of women's versus men's share in manufacturing	0.0187*	0.00487	0.00091	0.00303	0.00177	0.00073	0.00043**
Czechia & Poland	Exposure to robots	0.00195	0.00041	− 0.00436	0.00246	0.00178**	− 0.00013	− 0.000023
	Exposure to robots # Initial share of workers out of manufacturing	− 0.00007	− 0.00003*	0.00005*	− 0.00004	− 0.00003***	0.000000	− 0.00000
	Exposure to robots # Initial ratio of women's versus men's share in manufacturing	0.00402	0.00228***	0.00099	0.0003	0.00063	0.00014	0.00002

***1% **5% *10%. Sample sizes: 680 observations for Germany, 440 for France, 400 for Italy, 700 for the UK, and 240 for Poland and Czechia jointly.

The findings for the UK and Czechia and Poland are more consistent with our expectations. In the UK, the interaction between exposure to robotisation and the ratio of women’s and men’s employment in manufacturing is positive at all reproductive ages and significant in the models for the total fertility. At the same time, the effect of robot adoption on fertility in highly industrialised regions where employment in manufacturing is dominated by men is negative, suggesting that robotisation reduces fertility in such regions. In Czechia and Poland, the interaction between exposure to robotisation and ratio of women’s and men’s employment in manufacturing is positive at all reproductive ages (like in the UK), but significant only at ages 20–24.

### Educational Attainment of the Population

Subsequently, we test the hypothesis that robots exert a more negative impact on fertility in lower educated regions (H3). We present our findings in Table 4 where the rows 'Exposure to robots' denote the fertility effects of robotisation in regions with low educated populations and the interaction term demonstrates how these effects differ across regions with an increase in the proportion of educated persons by 1 pp.

We find clear support for hypothesis H3 in Germany and Italy. There is some evidence for this hypothesis also in the remaining countries but for France where our findings suggest the opposite.

**Table 4:** Exposure to robots ($\alpha$) and its interaction with the share of the highly educated population (ISCED 5–8)

Country	Measure	TFR	FR 20–24	FR 25–29	FR 30–34	FR 35–39	FR 40–44	FR 45 +
Germany	Exposure to robots	− 0.00161***	− 0.00027*	− 0.00011	− 0.00045**	− 0.00044***	− 0.00014***	− 0.00001**
	Exposure to robots # Share of highly educated	0.00005***	0.00001**	0.00001	0.00002**	0.00001***	0.000003**	0.0000003**
France	Exposure to robots	0.0015**	0.00058**	0.00105***	0.00019	− 0.00027	− 0.00015**	− 0.00001
	Exposure to robots # Share of highly educated	− 0.000054**	− 0.00002***	− 0.00003**	− 0.00000	0.00001	0.000004*	0.000000
Italy	Exposure to robots	− 0.00292*	− 0.00102	− 0.00124**	0.0002	− 0.00016	− 0.0002*	0.00001
	Exposure to robots # Share of highly educated	0.0001	0.00004*	0.00002	− 0.00001	0.00002	0.00001**	− 0.00000
UK	Exposure to robots	0.00026	− 0.00049	0.00171*	0.00063	0.00008	− 0.00016	− 0.00009**
	Exposure to robots # Share of highly educated	0.00003	− 0.000009	− 0.00002	0.00001	0.00002	0.00001	0.000002***
Czechia & Poland	Exposure to robots	− 0.00018	0.00039	− 0.00182***	0.00023	0.00066***	0.00002	− 0.00003***
	Exposure to robots # Share of highly educated	0.000021	− 0.00002	0.00007**	0.00001	− 0.00002**	− 0.00000	0.000001**

***1% **5% *10%. Sample sizes: 680 observations for Germany, 440 for France, 400 for Italy, 700 for the UK, and 240 for Poland and Czechia jointly.

In Germany, we identify a significantly negative effect of exposure to robots on fertility in regions characterised by lower educational attainment of the population: An increase in the exposure to robotisation by 1 robot per 10,000 workers leads to a decline in total fertility by 0.0016 there. Negative and mostly significant fertility effects are found at all reproductive ages. They clearly weaken with an increase in the proportion of highly educated individuals in a region. We find some traces of a similar pattern in Italy and Czechia and Poland, but the estimated effects are significant only at some ages and in Czechia and Poland some reversed findings are also obtained for the age group 35–39. The educational attainment of the regional population does not seem to matter for the effects of robotisation on fertility in the UK (except for highest reproductive ages where the findings are consistent with our expectations). Finally, in France we find that robotisation has a positive influence on fertility in regions with fairly low educated populations, which is in contrast to our hypothesis H3.

### Region’s Orientation at Investments in Knowledge and Technology

Finally, we expected the fertility effects of robotisation to be less negative or more positive in regions which are better able to embrace technological change. We operationalise this ability with the regional investment in technology- and knowledge-intensive sectors, measured by its employment. Only a few findings are consistent with this hypothesis (Table 5).

**Table 5:** Exposure to robots ($\alpha$) and its interaction with the share of workers employed in technology- and knowledge-intensive sectors

Country	Measure	TFR	FR 20–24	FR 25–29	FR 30–34	FR 35–39	FR 40–44	FR 45 +
Germany	Exposure to robots	− 0.00006	0.0001	0.00015**	− 0.00003	− 0.00015***	− 0.00005***	− 0.000003*
	Exposure to robots # Share employed in technology and knowledge sectors	− 0.00002	− 0.00001	− 0.00005***	0.00001	0.00002*	0.00001	0.000001**
France	Exposure to robots	− 0.00015	− 0.00019*	0.00006	0.00013	− 0.00004	− 0.00007***	− 0.000002
	Exposure to robots # Share employed in technology and knowledge sectors	0.00007	0.00003	0.00002	− 0.00000	0.00001	0.00001*	0.000002
Italy	Exposure to robots	− 0.00116*	− 0.00013	− 0.00117***	− 0.00017	0.00037***	− 0.00001	0.000002
	Exposure to robots # Share employed in technology and knowledge sectors	0.000005	− 0.00002	0.0001	0.00002	− 0.00008	− 0.00001	0.000002
UK	Exposure to robots	0.00161	− 0.0008	0.00122	0.00151	0.00071	0.00016	− 0.00001
	Exposure to robots # Share employed in technology and knowledge sectors	0.00001	0.00000	− 0.00020*	− 0.00005	0.00012	0.00007*	− 0.00000
Czechia & Poland	Exposure to robots	0.00119	0.00025	− 0.00047	0.00096	0.00039*	− 0.00009**	− 0.00003***
	Exposure to robots # Share employed in technology and knowledge sectors	− 0.00031	− 0.00004	− 0.00003	− 0.00022	− 0.00006	0.00002	0.000006**

***1% **5% *10%. Sample sizes: 680 observations for Germany, 440 for France, 400 for Italy, 700 for the UK, and 240 for Poland and Czechia jointly.

On the one hand, we find the interaction term between exposure to robotisation and employment in technology- and knowledge-intensive sectors to be significantly negative at lower reproductive ages (25–29) in Germany and the UK. On the other hand, however, the interaction term turns often positive and significant at high reproductive ages. This latter finding emerges clearly in Germany, but also to a lower extent in France, UK and Czechia and Poland, suggesting fertility recuperation (or higher-order fertility) encouraged by increasing employment/earning opportunities and growing prosperity of the region.

## Discussion

Industrial robots substantially change the conditions of participating in the labour markets and thereby may also affect fertility. On the one hand, there is evidence that robots destroy jobs, increase turnover in the labour market and make workers adjust to the new demands in the labour markets (reskill, upskill or increase work effort to follow the new work guidelines or even keep the job). On the other hand, however, robots may also increase productivity and thereby contribute to the expansion of new jobs, in particular in regions with highly educated workforce open to technological innovations. In this study, we examined whether these long-term structural changes, driven by adoption of industrial robots, affect regional fertility rates in six European countries. We find that fertility effects of robot adoption are rather small and vary across regions, depending on workforce education, employment structure and region’s capacity to embrace technological change. Briefly, our findings suggest that robots tend to exert a negative influence on fertility in regions where substantial numbers of workers are exposed to losing their jobs due to automation, i.e. highly industrialised regions (except for Czechia and Poland) and regions with relatively low educated populations (except for France). We also find the fertility effects to be more negative in less technologically advanced regions where robotisation is unlikely to boost productivity and create new jobs. The negative fertility effects are clearly most evident at young ages, especially in regions with large manufacturing sectors and to some extent in regions with lower educated populations. This finding may suggest postponement of fertility to higher ages, though fertility recuperation at older ages does not emerge clearly from our study, except for regions which are strongly oriented at knowledge and technological innovations. These findings are consistent with past research, showing that highly educated individuals, whose skills are valued in the labour market, tend to postpone childbearing into higher ages (Kantorova, 2004; Neels & De Wachter, 2010), but tend to recuperate it so that educational differences in cohort fertility tend to be smaller or even disappear in better developed regions (Nisen et al. 2021).

We also observe some country differences in fertility effects of robot adoption, but the pattern is not very clear. We see the negative effects of robots on fertility to be most pronounced in Germany, which is most advanced in automation among the studied countries. This is despite the strong employment protection in the country. We also observe some negative effects in Italy and less so in the UK. Robotisation in these two countries has progressed more slowly than in Germany, but employment protection is weaker there (in Italy low protection concerns disproportionately the young workers) and support for the unemployed is more limited. We also find the effects of robot adoption to be less disruptive for fertility and even to encourage it in Czechia and Poland. This finding is seemingly striking, but we explain it by the fact that robots are less likely to replace labour in countries with lower labour costs (Bachmann et al., 2022; Jung & Lim, 2020), which Czechia and Poland undoubtedly are in comparison with the Western European states. Moreover, we are puzzled by the fact that consistently with hypothesis H2 we find less negative effects of robot adoption in those British, Polish and Czech regions—where the ratio of women’s to men’s initial employment in manufacturing was higher—but not in Germany, France or Italy, even though the division of paid work between partners in Germany or Italy is not less asymmetric than in Poland or the UK (Matysiak & Steinmetz, 2008; Matysiak & Vignoli, 2013). One possible explanation for this finding might be related to the fact that women working in manufacturing moved out into the service sector much more quickly than men. Such a phenomenon was indeed observed in countries most advanced in automation (Black & Spitz-Oener, 2010; Cortes et al., 2021), which Germany, Italy and France indeed are. At the same time, the new jobs in the service sector turned out to be characterised by high insecurity and precarity with employers requiring from workers great deal of flexibility (Allen & Henry, 1997; Reimer, 1998). Finally, we find robotisation to exert most negative impact on fertility in regions with low-educated population in all analysed countries except for France. Past studies indeed showed that education is a weaker predictor of the realisation of fertility intentions in France than in Italy (Régnier-Loilier and Vignoli 2011) and that economic uncertainty is less disruptive for fertility in France than in Germany (Salles et al., 2016), likely because of the strong two-child family norm in France, less pronounced specialisation of partners in paid and unpaid labour and generous financial transfers to families, including the unemployment schemes (Pailhé and Solaz 2012). For these reasons, the French may be less sensitive to the risks resulting from long-term developments in the labour markets than other nations we studied. There is no doubt, however, that more in-depth insights are needed into the topic to corroborate our interpretations.

Our study is not without limitations. Due to the anonymisation procedures at Eurostat, some of our data were missing and had to be imputed. As a result our main measure, exposure to robots, contains measurement error, which causes its increased variance in comparison with a perfect measurement. Thus, we expect all regression lines that we fitted to be biased towards 0 (regression dilution/attenuation; Fuller, 1987). Our measure of exposure to robotisation faces other problems as well. Although it is at the forefront of economic research on automation and employment (Acemoglu & Restrepo, 2020; Dauth et al., 2021), it assumes that regional employment structure by sector remains unchanged over time. This assumption is needed in order to keep exposure to robots exogeneous, as the regional employment shares by sector are measured before the start of robotisation. Furthermore, we were not able to include more countries into our study. The adopted instrumental variable strategy, which implied instrumenting robotisation in one European country with robot adoption in other European countries, left us with no possibility to pool all European countries. Comparing a greater number of countries was not feasible since we had to choose countries with a reasonably large number of NUTS-2 regions. Last but not least, our analytical strategy did not allow us to account for possible spatial spillovers which may take place if workers commute to jobs outside of the regions of their residence (Monte et al., 2018). According to our best knowledge, in econometric literature exploiting sectoral composition as a source of local labour demand shocks (Bartik shocks) and in particular discussing the exposure to robots, no solutions to the two above-mentioned issues have been offered so far. We underline them as important areas for future research.

Despite these limitations and some inconsistencies, our findings suggest that long-term structural changes, driven by automation, can indeed affect fertility as it was proposed by Seltzer (2019). Nonetheless, it does not seem robotisation is primarily responsible for fertility declines observed in the aftermath of the Great Recession in most advanced countries. It exerts a negative influence on fertility in certain regions (highly industrialised or low/middle educated), but these effects are compensated by fertility increases in better educated and dynamically developing regions. It is likely that fertility is also affected by other components of structural labour market changes, driven by digitalisation, such as implementation of digital automats which also replace workers but are not classified as industrial robots, spread of remote work or increasingly widespread use of AI. Another possibility is that our study, conducted at the macro level, masks some important nuances such as differential effects of automation on workers’ fertility. These effects may certainly differ by workers’ gender and socio-economic status (education or occupation) or firm characteristics (firm’s capacity to retrain and retain workers). Fertility effects of automation may also depend on the labour market situation of the other partner and whether he or she is affected by automation as well. Future research should thus account for other aspects of long-term structural changes in the labour market, besides automation, and involve individual-level data in order to look more closely into specific circumstances of workers. More research is also needed to unravel the mechanisms which underlie these relationships. Several mechanisms are possible, among them certainly job displacement, job-related uncertainty or pressure to reskill and adapt to new work guidelines and ways of working. Finally, future research should more closely explore the cross-country differences in fertility effects of long-term labour market changes caused, among others, by automation. In particular, it is of vital importance to understand which specific public policies and other institutional factors may mitigate the negative consequences of automation on fertility. Being one of the first attempts to investigate the role of labour market changes, driven by automation, for fertility this single study is not able to address all these questions but certainly aims at stimulating future research on the topic.

### Electronic supplementary material

Below is the link to the electronic supplementary material. Tables 1-20 (located in the Word file) present full results from interaction IV models presented in the paper. They supplement the basic models presented in the Appendix. Tables 21-45 in Excel present full results from the basic and interaction OLS models.

Supplementary file1 (DOCX 57 KB)

Supplementary file2 (XLSX 660 KB)

## Appendix

### Reclassifying Industry

The regional employment structure data are aggregates obtained from the European Union Labour Force Survey microdata. We reclassify them to 16 ISIS categories that we operationalise for the robot data using the correspondence table available through the online resources of the United Nations Statistics Division (see Table 6). As can be seen in the table, in some cases, it involves summing employment for 2 or 3 NACE categories to match the ISIC category.

**Table 6:** ISIC-NACE industry codes crosswalk for sectors used in our analysis

Category	IFR (ISIC)	Regional employment (na112d)	Regional employment (nace2d)
All other manufacturing branches/other chemical products n.e.c	91, 20–21	30, 37, 23	32, 33, 19
Automotive/other vehicles	29–30	34, 35	29, 30
Basic metals	24	27	24
Construction	F	45	41, 42, 43
Electrical/electronics	26–27	31, 32, 33	26, 27
Electricity, gas, water supply	E	40, 41	35, 36
Food and beverages	10–12	15, 16	10, 11, 12
Glass, ceramics, stone, mineral products (non-automotive)	23	26	23
Industrial machinery	28	29	28
Metal products (non-automotive)	25	28	25
Mining and quarrying	C	10, 11, 12, 13, 14	05, 06, 07, 08, 09
Paper	17–18	21, 22	17, 18
Pharmaceuticals, cosmetics	19	24	20, 21
Rubber and plastic products (non-automotive)	22	25	22
Textiles	13–15	17, 18, 19	13, 14, 15
Wood and furniture	16	20, 36	16, 31

**Table 7:** NUTS-2 region splits/merges over years (1994–2017)

1994–1998	1999	2000–2001	2002–2003	2004	2005–2010	2011–2012	2013–2017	Action
DE40	DE40	DE40	DE40	DE41	DE40	DE40	DE40	sum DE41 and DE42 to DE40
				DE42				
DEB1	DEB1	DEB0	DEB1	DEB1	DEB1	DEB1	DEB1	sum DEB1, DEB2, and DEB3 to DEB0
DEB2	DEB2		DEB2	DEB2	DEB2	DEB2	DEB2	
DEB3	DEB3		DEB3	DEB3	DEB3	DEB3	DEB3	
DED0	DED0	DED2	DED2	DED2	DED2	DED2	DED2	sum DED2, DED4, and DED5 to DED0
		DED4	DED4	DED4	DED4	DED4	DED4	
		DED5	DED5	DED5	DED5	DED5	DED5	
DEE1	DEE1	DEE1	DEE1	DEE1	DEE0	DEE0	DEE0	sum DEE1, DEE2, and DEE3 to DEE0
DEE2	DEE2	DEE2	DEE2	DEE2				
DEE3	DEE3	DEE3	DEE3	DEE3				
IT31	ITH1	ITH1	ITH1	ITH1	ITH1	ITH1	ITH1	sum ITH1 and ITH2 to IT31
	ITH2	ITH2	ITH2	ITH2	ITH2	ITH2	ITH2	
UKI1	UKI1	UKI1	UKI1	UKI1	UKI1	UKI3	UKI3	sum UKI3 and UKI4 to UKI1
						UKI4	UKI4	
UKI2	UKI2	UKI2	UKI2	UKI2	UKI2	UKI5	UKI5	sum UKI5, UKI6, and UKI7 to UKI2
						UKI6	UKI6	
						UKI7	UKI7	
PL12	PL12	PL12	PL12	PL12	PL12	PL12	PL91	sum PL91 and PL92 to PL12
							PL92

**Table 8:** Full basic model results for Germany (see Table 1 in Sect. 6.1)

Covariate	TFR	FR 20–24	FR 25–29	FR 30–34	FR 35–39	FR 40–44	FR 45 +
Exposure to robots	− 0.000159	0.0000438	0.0000217	− 0.0000215	− 0.000110***	− 0.0000484***	− 0.00000125
Share of population aged 15–24	− 1.141**	− 0.821***	− 0.424***	0.710***	− 0.179**	− 0.164***	− 0.0135***
Share of population aged 25–49	2.184*	− 0.0586	− 0.579	1.537***	0.978***	0.0514	− 0.0156
Share of population aged 50 +	0.522	− 0.257*	− 0.430**	1.106***	0.141	− 0.167***	− 0.0225***
Share of highly educated population	− 0.00173	− 0.00133**	− 0.00335***	0.000463	0.00182***	0.000682***	0.0000721***
Ratio of share of highly educated women to share of highly educated men	− 0.651***	− 0.0780**	− 0.140**	− 0.223***	− 0.127***	− 0.021	− 0.00263*
Square of ratio of share of highly educated women to highly educated men	0.522***	0.0711***	0.124***	0.161***	0.0980***	0.0183***	0.00183**
Share of economically active women	− 0.000723	− 0.000145	0.00125*	− 0.000427	− 0.000933*	− 0.000450**	− 0.0000538***
Kleibergen-Paap rk Wald F statistic	347.778	347.778	347.778	347.778	347.778	347.778	347.778
Hansen J p-value	0.4002	0.3592	0.4523	0.0432	0.0281	0.2845	0.2264

^***^1% **5% *10%. *N* = 680, 20 years, 34 NUTS2 regions. Further controls include yearly dummies (partialled out). Standard errors are clustered at region level

**Table 9:** Full basic model results for France (see Table 1 in Sect. 6.1)

Covariate	TFR	FR 20–24	FR 25–29	FR 30–34	FR 35–39	FR 40–44	FR 45 +
Exposure to robots	0.000026	− 0.000102	0.0000935	0.000118	− 0.0000115	− 0.0000356	0.00000348
Share of population aged 15–24	− 3.278	− 3.082***	− 0.0862	0.299	0.34	0.0384	0.026
Share of population aged 25–49	− 7.505***	− 3.952***	− 1.944**	− 0.307	− 0.212	− 0.189	0.00224
Share of population aged 50 +	− 6.268***	− 2.929***	− 1.067*	− 0.579	− 0.645***	− 0.336***	− 0.0457***
Share of highly educated population	0.00187	0.000263	0.00035	0.000389	0.000697**	0.000121	0.0000129
Ratio of share of highly educated women to share of highly educated men	0.223	− 0.0326	− 0.106	0.17	0.147**	0.0642**	0.00710**
Square of ratio of share of highly educated women to highly educated men	− 0.109	0.0116	0.0409	− 0.0771*	− 0.0637***	− 0.0274**	− 0.00259**
Share of economically active women	− 0.00113	0.000105	0.000387	− 0.000880*	− 0.000793***	− 0.000354***	− 0.00000468
Kleibergen-Paap rk Wald F statistic	1042.809	1042.809	1042.809	1042.809	1042.809	1042.809	1042.809
Hansen J p-value	0.6166	0.2884	0.3651	0.4868	0.4660	0.1540	0.8730

^***^1% **5% *10%. *N* = 440, 20 years, 22 NUTS2 regions. Further controls include yearly dummies (partialled out). Standard errors are clustered at region level

**Table 10:** Full basic model results for Italy (see Table 1 in Sect. 6.1)

Covariate	TFR	FR 20–24	FR 25–29	FR 30–34	FR 35–39	FR 40–44	FR 45 +
Exposure to robots	− 0.00118*	− 0.000196	− 0.000898***	− 0.000116	0.00014	− 0.0000473	0.00000823
Share of population aged 15–24	− 9.167***	− 4.185***	− 3.894***	0.368	− 0.686	− 0.461***	− 0.0355*
Share of population aged 25–49	− 7.001***	− 1.823***	− 4.355***	− 0.810***	0.14	− 0.167*	− 0.02
Share of population aged 50 +	− 6.623***	− 2.395***	− 3.063***	− 0.321*	− 0.373**	− 0.264***	− 0.0108
Share of highly educated population	− 0.00181	− 0.000107	− 0.00257**	− 0.00181*	0.000255	0.00118***	0.000136*
Ratio of share of highly educated women to highly educated men	− 0.0448	− 0.109	− 0.0746	0.105	0.0448	− 0.0258	− 0.00212
Square of ratio of share of highly educated women to highly educated men	0.0536	0.0511*	0.0308	− 0.0308	− 0.00361	0.0113	0.000552
Share of economically active women	0.00296	0.00197***	0.000202	− 0.000201	0.00083	0.0000921	0.0000639
Kleibergen-Paap rk Wald F statistic	175.284	175.284	175.284	175.284	175.284	175.284	175.284
Hansen J p-value	0.2683	0.3285	0.1599	0.7500	0.1742	0.5438	0.3200

^***^1% **5% *10%. *N* = 400, 20 years, 20 NUTS2 regions. Further controls include yearly dummies (partialled out). Standard errors are clustered at region level

**Table 11:** Full basic model results for the UK (see Table 1 in Sect. 6.1)

Covariate	TFR	FR 20–24	FR 25–29	FR 30–34	FR 35–39	FR 40–44	FR 45 +
Exposure to robots	0.00168	− 0.000872	0.000793	0.00133	0.00109	0.000386*	− 0.00000172
Share of population aged 15–24	0.555	− 2.063***	0.595	2.364***	1.35	0.16	− 0.0840***
Share of population aged 25–49	− 0.491	− 1.086*	− 0.437	1.422	0.803	− 0.0106	− 0.0516*
Share of population aged 50 +	2.041	0.0326	0.372	1.462**	0.918	0.032	− 0.0430*
Share of highly educated population	0.000193	0.000994	− 0.000392	− 0.00058	− 0.000765**	− 0.000104	0.0000304*
Ratio of share of highly educated women to share of highly educated men	1.032***	0.213*	0.241*	0.367***	0.224**	0.0436	-0.000287
Square of ratio of share of highly educated women to highly educated men	− 0.490***	− 0.107*	− 0.105	− 0.169***	− 0.110***	− 0.0223	− 0.0000613
Share of economically active women	− 0.00260*	− 0.000123	− 0.000653	− 0.000624	− 0.000863**	− 0.000277**	− 0.0000203
Kleibergen-Paap rk Wald F statistic	137.303	137.303	137.303	137.303	137.303	137.303	137.303
Hansen J p-value	0.0363	0.0847	0.6383	0.0140	0.0815	0.1513	0.0684

^***^1% **5% *10%. *N* = 700, 20 years, 35 NUTS2 regions. Further controls include yearly dummies (partialled out). Standard errors are clustered at region level

**Table 12:** Full basic model results for Poland and Czechia (see Table 1 in Sect. 6.1)

Covariate	TFR	FR 20–24	FR 25–29	FR 30–34	FR 35–39	FR 40–44	FR 45 +
Exposure to robots	0.000530	0.000104	− 0.000436	0.000501	0.000253*	− 0.0000469	− 0.0000119
Share of population aged 15–24	− 1.043	− 0.893	0.506	2.092***	− 1.640***	− 0.801***	− 0.0889***
Share of population aged 25–49	− 6.482**	− 2.873***	− 1.501	1.013	− 1.432***	− 0.683***	− 0.0819***
Share of population aged 50 +	− 3.253***	− 1.658***	− 0.478	1.379**	− 1.273***	− 0.616***	− 0.0698***
Share of highly educated population	0.00325	− 0.00108	0.000824	0.00320***	0.000581	− 0.000123	0.0000267
Ratio of share of highly educated women to share of highly educated men	0.151	0.0122	− 0.263***	0.166	0.171**	0.0228	0.00496*
Square of ratio of share of highly− educated women to highly educated men	− 0.0445	− 0.00413	0.104***	− 0.0581	− 0.0580**	− 0.00915	− 0.00182*
Share of economically active women	0.00692**	0.00185	0.00518***	0.000411	− 0.000553	0.0000953	− 0.0000223
Kleibergen-Paap rk Wald F statistic	45.992	45.992	45.992	45.992	45.992	45.992	45.992
Hansen J p-value	0.1429	0.0456	0.0299	0.2654	0.1859	0.1341	0.1430

^***^1% **5% *10%. *N* = 240, 10 years, 24 NUTS2 regions. Further controls include yearly dummies (partialled out). Standard errors are clustered at region level

### Imputing Regional Employment Structures

Eurostat anonymises records where employment in a specific region, industry, and year was above zero but below 1,000 people, i.e. information is missing for such records. As a result, 50% of employment records were initially missing in the data. In the cases when only observations for specific years for a given region-industry are missing, we impute it by drawing a number between 0 and 1000 from a uniform distribution. In the cases when the entire time series for a given region-industry is missing, we impute it with median employment for that industry in the country, normalised to a 0–1000 range. Since our main explanatory measure (described in detail in the Sect. 5.2 in the main text) relies on a sum of employment over industries, it would be impossible to construct it without assumptions about the missing data. We decided to choose the imputation with median instead of the mean, to robustify the imputed data to extreme values in existing data. One should bear in mind that, after imputation, there is a measurement error in our regional employment data. Thus, the regression coefficient corresponding to our main measure will be downward-biased (regression dilution bias; Fuller, 1987).

### Reclassifying NUTS 2 Codes

The NUTS classification of regions underwent a few reclassifications in its history. Eurostat usually publishes regional data for specific years for regions which were operative depending on then-current NUTS classification. To obtain a balanced panel, we reclassify all regional codes, which simply changed name, to the NUTS 2016 classification, using crosswalks available on the Eurostat web page. For the countries and time frame we consider in our analysis, there are eight cases when two or three regions split or merged resulting in changes in the NUTS classification (see Table 7). In those instances, we sum up/average (depending on a variable) data for the smaller regions to obtain consistent data for the larger region. We exclude 5 French overseas territories with distinct socioeconomic setups, not directly comparable to European regions (Guadeloupe, Martinique, French Guiana, La Reunion, and Mayotte).

## Footnotes

1 Automotive/Other vehicles, basic metals, electrical/electronics, food and beverages, glass, ceramics, stone, mineral products (non-automotive), industrial machinery, metal products (non-automotive), paper, pharmaceuticals, cosmetics, rubber and plastic products (non-automotive), textiles, wood and furniture, all other manufacturing branches/other chemical products not elsewhere classified.
