# Supplementary material for: Industrial Robots and Regional Fertility in European Countries
Source: Eur J Popul. 2023 Mar 28;39(1):11. doi: 10.1007/s10680-023-09657-4 (PMC10043858; doi:10.1007/s10680-023-09657-4)
Supplement: Supplementary file 1 — Supplementary file1 (DOCX 57 KB) [file 10680_2023_9657_MOESM1_ESM.docx]

**Online Supplementary Material**

Tables 1-20 present full results from interaction IV models presented in the paper. They supplement the basic models presented in the Appendix.

Tables 21-45 in Excel present full results from the basic and interaction OLS models.

Table 1. IV: Full interaction model results for Germany (see Table 2 in Section 6.2). Interaction of exposure to robots with the initial share of workers out of manufacturing.

| Covariate | TFR | FR 20-24 | FR 25-29 | FR 30-34 | FR 35-39 | FR 40-44 | FR 45+ |
| --- | --- | --- | --- | --- | --- | --- | --- |
| Exposure to robots | -0.00220** | -0.00121*** | -0.00137*** | 0.000373 | 0.000085 | -0.0000287 | -0.00000116 |
| Exposure to robots # Initial share of workers out of manufacturing | 0.0000287** | 0.0000176*** | 0.0000195*** | -5.39E-06 | -0.00000269 | -2.61E-07 | -2.46E-09 |
| Share of population aged 15-24 | -0.970* | -0.716*** | -0.307** | 0.678*** | -0.195** | -0.166*** | -0.0136*** |
| Share of population aged 25-49 | 2.222* | -0.044 | -0.564* | 1.544*** | 0.980*** | 0.0525 | -0.0157 |
| Share of population aged 50+ | 0.732 | -0.134 | -0.293 | 1.075*** | 0.124 | -0.168*** | -0.0226*** |
| Share of highly educated population | -0.00101 | -0.00088 | -0.00285*** | 0.000306 | 0.00174*** | 0.000673*** | 0.0000722*** |
| Ratio of share of highly-educated women to share of highly-educated men | -0.646*** | -0.0742** | -0.136** | -0.225*** | -0.128*** | -0.0211 | -0.00262* |
| Square of ratio of share of highly-educated women to share of highly-educated men | 0.488*** | 0.0505** | 0.101*** | 0.168*** | 0.101*** | 0.0186** | 0.00183** |
| Share of economically active women | -0.00115 | -0.000417 | 0.000951 | -0.00033 | -0.000887* | -0.000444** | -0.0000539*** |
| Kleibergen-Paap rk Wald F statistic | 766.125 | 766.125 | 766.125 | 766.125 | 766.125 | 766.125 | 766.125 |
| Hansen J p-value | 0.1929 | 0.0330 | 0.0706 | 0.1691 | 0.1063 | 0.4316 | 0.2074 |

*** 1% ** 5% * 10%. N=680, 20 years, 34 NUTS2 regions. Further controls include yearly dummies (partialled out). Standard errors are clustered at region level.

Table 2. IV: Full interaction model results for France (see Table 2 in Section 6.2). Interaction of exposure to robots with the initial share of workers out of manufacturing.

| Covariate | TFR | FR 20-24 | FR 25-29 | FR 30-34 | FR 35-39 | FR 40-44 | FR 45+ |
| --- | --- | --- | --- | --- | --- | --- | --- |
| Exposure to robots | 0.00163 | 0.000616 | 0.00212 | 0.000125 | -0.000815 | -0.000453** | -0.0000814* |
| Exposure to robots # Initial share of workers out of manufacturing | -0.0000231 | -0.0000102 | -0.0000289 | -1.85E-07 | 0.0000114 | 0.00000589* | 0.00000120* |
| Share of population aged 15-24 | -3.384 | -3.126*** | -0.205 | 0.294 | 0.383 | 0.0605 | 0.0307 |
| Share of population aged 25-49 | -7.723*** | -4.047*** | -2.212** | -0.31 | -0.109 | -0.135 | 0.0133 |
| Share of population aged 50+ | -6.429*** | -3.001*** | -1.272** | -0.579 | -0.563*** | -0.294*** | -0.0371*** |
| Share of highly educated population | 0.00191 | 0.000283 | 0.000402 | 0.000392 | 0.000679** | 0.000112 | 0.000011 |
| Ratio of share of highly-educated women to share of highly-educated men | 0.251 | -0.0215 | -0.0753 | 0.171 | 0.136** | 0.0586** | 0.00590* |
| Square of ratio of share of highly-educated women to share of highly-educated men | -0.12 | 0.00693 | 0.0282 | -0.0777 | -0.0592** | -0.0250** | -0.00209* |
| Share of economically active women | -0.00122 | 0.0000695 | 0.000292 | -0.000887* | -0.000762*** | -0.000338*** | -0.00000112 |
| Kleibergen-Paap rk Wald F statistic | 443.790 | 443.790 | 443.790 | 443.790 | 443.790 | 443.790 | 443.790 |
| Hansen J p-value | 0.5146 | 0.1284 | 0.1813 | 0.3136 | 0.2227 | 0.4156 | 0.8597 |

*** 1% ** 5% * 10%. N=440, 20 years, 22 NUTS2 regions. Further controls include yearly dummies (partialled out). Standard errors are clustered at region level.

Table 3. IV: Full interaction model results for Italy (see Table 2 in Section 6.2). Interaction of exposure to robots with the initial share of workers out of manufacturing.

| Covariate | TFR | FR 20-24 | FR 25-29 | FR 30-34 | FR 35-39 | FR 40-44 | FR 45+ |
| --- | --- | --- | --- | --- | --- | --- | --- |
| Exposure to robots | -0.00264 | -0.000509 | -0.00201 | -0.000852 | 0.000687 | 0.000390** | -0.000127** |
| Exposure to robots # Initial share of workers out of manufacturing | 0.0000216 | 0.00000471 | 0.0000157 | 0.0000104 | -0.00000734 | -0.00000598** | 0.00000186*** |
| Share of population aged 15-24 | -9.648*** | -4.294*** | -4.221*** | 0.155 | -0.551 | -0.346*** | -0.0720*** |
| Share of population aged 25-49 | -7.148*** | -1.855*** | -4.464*** | -0.882*** | 0.192 | -0.125 | -0.0330** |
| Share of population aged 50+ | -6.888*** | -2.454*** | -3.251*** | -0.445* | -0.289 | -0.194*** | -0.0326*** |
| Share of highly educated population | -0.00252 | -0.000274 | -0.00302*** | -0.00210** | 0.00041 | 0.00132*** | 0.0000898 |
| Ratio of share of highly-educated women to share of highly-educated men | -0.0113 | -0.102 | -0.0507 | 0.121* | 0.034 | -0.0347 | 0.000651 |
| Square of ratio of share of highly-educated women to share of highly-educated men | 0.0419 | 0.0485 | 0.0225 | -0.0362 | 0.0000231 | 0.0143* | -0.000397 |
| Share of economically active women | 0.00299 | 0.00198*** | 0.000197 | -0.000208 | 0.000852 | 0.000105 | 0.0000606 |
| Kleibergen-Paap rk Wald F statistic | 188.524 | 188.524 | 188.524 | 188.524 | 188.524 | 188.524 | 188.524 |
| Hansen J p-value | 0.2720 | 0.1686 | 0.1761 | 0.6730 | 0.4647 | 0.1289 | 0.2605 |

*** 1% ** 5% * 10%. N=400, 20 years, 20 NUTS2 regions. Further controls include yearly dummies (partialled out). Standard errors are clustered at region level.

Table 4. IV: Full interaction model results for the UK (see Table 2 in Section 6.2). Interaction of exposure to robots with the initial share of workers out of manufacturing.

| Covariate | TFR | FR 20-24 | FR 25-29 | FR 30-34 | FR 35-39 | FR 40-44 | FR 45+ |
| --- | --- | --- | --- | --- | --- | --- | --- |
| Exposure to robots | -0.0223** | -0.00584 | -0.000935 | -0.000879 | -0.00384 | -0.00155 | 0.000116 |
| Exposure to robots # Initial share of workers out of manufacturing | 0.000311** | 0.0000647 | 0.0000225 | 0.0000278 | 0.0000636 | 0.0000251 | -0.00000161 |
| Share of population aged 15-24 | 0.814 | -2.013*** | 0.613 | 2.400*** | 1.409 | 0.182 | -0.0841*** |
| Share of population aged 25-49 | -0.458 | -1.082* | -0.436 | 1.436 | 0.816 | -0.00704 | -0.0507* |
| Share of population aged 50+ | 2.117 | 0.0456 | 0.377 | 1.479** | 0.939 | 0.0391 | -0.0424* |
| Share of highly educated population | -0.000215 | 0.000912 | -0.000421 | -0.000624 | -0.000852*** | -0.000137 | 0.0000317* |
| Ratio of share of highly-educated women to share of highly-educated men | 1.100*** | 0.227* | 0.246* | 0.375*** | 0.239** | 0.0492 | -0.000415 |
| Square of ratio of share of highly-educated women to share of highly-educated men | -0.522*** | -0.114** | -0.107 | -0.173*** | -0.117*** | -0.025 | 0.000000392 |
| Share of economically active women | -0.00232 | -0.0000639 | -0.000633 | -0.000603 | -0.000809** | -0.000255* | -0.0000222 |
| Kleibergen-Paap rk Wald F statistic | 118.083 | 118.083 | 118.083 | 118.083 | 118.083 | 118.083 | 118.083 |
| Hansen J p-value | 0.0505 | 0.1054 | 0.4370 | 0.1379 | 0.1993 | 0.2458 | 0.1026 |

*** 1% ** 5% * 10%. N=700, 20 years, 35 NUTS2 regions. Further controls include yearly dummies (partialled out). Standard errors are clustered at region level.

Table 5. IV: Full interaction model results for Poland and Czechia (see Table 2 in Section 6.2). Interaction of exposure to robots with the initial share of workers out of manufacturing.

| Covariate | TFR | FR 20-24 | FR 25-29 | FR 30-34 | FR 35-39 | FR 40-44 | FR 45+ |
| --- | --- | --- | --- | --- | --- | --- | --- |
| Exposure to robots | 0.00627 | 0.00295*** | -0.00337** | 0.00275 | 0.00251*** | 0.0000358 | 0.00000680 |
| Exposure to robots # Initial share of workers out of manufacturing | -0.0000961* | -0.0000466*** | 0.0000472** | -0.0000392 | -0.0000368*** | -0.00000105 | -0.000000341 |
| Share of population aged 15-24 | -3.114* | -1.784*** | 1.329* | 1.092 | -2.324*** | -0.789*** | -0.0990*** |
| Share of population aged 25-49 | -7.636*** | -3.317*** | -1.134 | 0.383 | -1.763*** | -0.660*** | -0.0889*** |
| Share of population aged 50+ | -5.057*** | -2.442*** | 0.252 | 0.518 | -1.877*** | -0.608*** | -0.0785*** |
| Share of highly educated population | 0.00268 | -0.00129 | 0.000988 | 0.00287*** | 0.000427 | -0.000108 | 0.0000229 |
| Ratio of share of highly-educated women to share of highly-educated men | 0.0472 | -0.0415 | -0.206*** | 0.128 | 0.128* | 0.0206 | 0.00468* |
| Square of ratio of share of highly-educated women to share of highly-educated men | -0.00664 | 0.0155 | 0.0831*** | -0.0444 | -0.0423* | -0.00834 | -0.00172* |
| Share of economically active women | 0.00687** | 0.00181* | 0.00521*** | 0.000402 | -0.000579 | 0.0000925 | -0.0000223 |
| Kleibergen-Paap rk Wald F statistic | 113.238 | 113.238 | 113.238 | 113.238 | 113.238 | 113.238 | 113.238 |
| Hansen J p-value | 0.5701 | 0.2830 | 0.2720 | 0.5985 | 0.3052 | 0.2425 | 0.5084 |

*** 1% ** 5% * 10%. N=240, 10 years, 24 NUTS2 regions. Further controls include yearly dummies (partialled out). Standard errors are clustered at region level.

Table 6. IV: Full interaction model results for Germany (see Table 3 in Section 6.3). Interaction of exposure to robots with the initial share of workers employed out of manufacturing and interaction of exposure to robots with the initial ratio of women’s to men’s employment share in manufacturing.

| Covariate | TFR | FR 20-24 | FR 25-29 | FR 30-34 | FR 35-39 | FR 40-44 | FR 45+ |
| --- | --- | --- | --- | --- | --- | --- | --- |
| Exposure to robots | 0.000791 | -0.00041 | -0.000178 | 0.00064 | 0.000532 | 0.000000707 | 0.00000748 |
| Exposure to robots # Initial share of workers out of manufacturing | 0.00000794 | 0.0000119** | 0.0000114** | -7.27E-06 | -5.84E-06 | -0.000000475 | -6.29E-08 |
| Exposure to robots # Initial ratio of women's versus men's share in manufacturing | -0.00350*** | -0.000933* | -0.00141*** | -0.000308 | -0.000520** | -0.000035 | -0.00001 |
| Share of population aged 15-24 | -0.891* | -0.695*** | -0.274* | 0.685*** | -0.183** | -0.165*** | -0.0133*** |
| Share of population aged 25-49 | 2.960*** | 0.148 | -0.258 | 1.611*** | 1.088*** | 0.0587 | -0.0136 |
| Share of population aged 50+ | 1.117** | -0.0347 | -0.131 | 1.109*** | 0.18 | -0.165*** | -0.0215*** |
| Share of highly educated population | 0.000668 | -0.000426 | -0.00218*** | 0.00045 | 0.00199*** | 0.000692*** | 0.0000770*** |
| Ratio of share of highly-educated women to share of highly-educated men | -0.558*** | -0.0505 | -0.101** | -0.217*** | -0.115*** | -0.0201 | -0.00237 |
| Square of ratio of share of highly-educated women to share of highly-educated men | 0.426*** | 0.0338 | 0.0755*** | 0.162*** | 0.0920*** | 0.0179** | 0.00165* |
| Share of economically active women | -0.00279 | -0.000858* | 0.000298 | -0.000472 | -0.00113** | -0.000462** | -0.0000586*** |
| Kleibergen-Paap rk Wald F statistic | 1091.572 | 1091.572 | 1091.572 | 1091.572 | 1091.572 | 1091.572 | 1091.572 |
| Hansen J p-value | 0.2375 | 0.1597 | 0.2198 | 0.2598 | 0.2384 | 0.4486 | 0.2395 |

*** 1% ** 5% * 10%. N=680, 20 years, 34 NUTS2 regions. Further controls include yearly dummies (partialled out). Standard errors are clustered at region level.

Table 7. IV: Full interaction model results for France (see Table 3 in Section 6.3). Interaction of exposure to robots with the initial share of workers employed out of manufacturing and interaction of exposure to robots with the initial ratio of women’s to men’s employment share in manufacturing.

| Covariate | TFR | FR 20-24 | FR 25-29 | FR 30-34 | FR 35-39 | FR 40-44 | FR 45+ |
| --- | --- | --- | --- | --- | --- | --- | --- |
| Exposure to robots | 0.0049 | 0.00188* | 0.00352** | 0.000801 | -0.000976* | -0.000558** | -0.000122** |
| Exposure to robots # Initial share of workers out of manufacturing | -0.0000207 | -0.00000593 | -0.0000278 | -1.09E-06 | 0.00001 | 0.00000476* | 0.000000961* |
| Exposure to robots # Initial ratio of women's versus men's share in manufacturing | -0.00681** | -0.00307*** | -0.00292* | -0.00122 | 0.000492 | 0.000357 | 0.000112** |
| Share of population aged 15-24 | -3.816 | -3.298*** | -0.391 | 0.207 | 0.407 | 0.0764 | 0.0365** |
| Share of population aged 25-49 | -8.312*** | -4.279*** | -2.465*** | -0.43 | -0.0775 | -0.114 | 0.0209 |
| Share of population aged 50+ | -6.641*** | -3.077*** | -1.363** | -0.626 | -0.554*** | -0.289*** | -0.0348*** |
| Share of highly educated population | 0.00189 | 0.000259 | 0.000392 | 0.000393 | 0.000685** | 0.000117 | 0.0000121 |
| Ratio of share of highly-educated women to share of highly-educated men | 0.209 | -0.0463 | -0.0928 | 0.166 | 0.141** | 0.0626** | 0.00695* |
| Square of ratio of share of highly-educated women to share of highly-educated men | -0.103 | 0.017 | 0.0353 | -0.0757 | -0.0612** | -0.0267** | -0.00252* |
| Share of economically active women | -0.00146 | -0.0000106 | 0.00019 | -0.000939* | -0.000753*** | -0.000333*** | 0.00000123 |
| Kleibergen-Paap rk Wald F statistic | 1690.910 | 1690.910 | 1690.910 | 1690.910 | 1690.910 | 1690.910 | 1690.910 |
| Hansen J p-value | 0.5372 | 0.4071 | 0.3465 | 0.5274 | 0.3034 | 0.6399 | 0.3241 |

*** 1% ** 5% * 10%. N=440, 20 years, 22 NUTS2 regions. Further controls include yearly dummies (partialled out). Standard errors are clustered at region level.

Table 8. IV: Full interaction model results for Italy (see Table 3 in Section 6.3). I Interaction of exposure to robots with the initial share of workers employed out of manufacturing and interaction of exposure to robots with the initial ratio of women’s to men’s employment share in manufacturing.

| Covariate | TFR | FR 20-24 | FR 25-29 | FR 30-34 | FR 35-39 | FR 40-44 | FR 45+ |
| --- | --- | --- | --- | --- | --- | --- | --- |
| Exposure to robots | 0.0144*** | 0.00535*** | 0.00670*** | -0.000561 | 0.000377 | 0.00116*** | -0.000144* |
| Exposure to robots # Initial share of workers out of manufacturing | -0.000113** | -0.0000422** | -0.0000539*** | 9.85E-06 | -4.66E-06 | -0.0000121*** | 0.00000195** |
| Exposure to robots # Initial ratio of women's versus men's share in manufacturing | -0.0137*** | -0.00462*** | -0.00693*** | -0.000394 | 0.000249 | -0.000605*** | 0.0000188 |
| Share of population aged 15-24 | -9.652*** | -4.258*** | -4.167*** | 0.0656 | -0.566 | -0.344** | -0.0695*** |
| Share of population aged 25-49 | -7.275*** | -1.887*** | -4.520*** | -0.907*** | 0.193 | -0.129 | -0.0322** |
| Share of population aged 50+ | -7.050*** | -2.489*** | -3.310*** | -0.494** | -0.291* | -0.200*** | -0.0311*** |
| Share of highly educated population | -0.00482 | -0.000992 | -0.00407*** | -0.00232** | 0.000416 | 0.00122*** | 0.000097 |
| Ratio of share of highly-educated women to share of highly-educated men | 0.0518 | -0.0827 | -0.0215 | 0.128* | 0.0334 | -0.0321 | 0.000403 |
| Square of ratio of share of highly-educated women to share of highly-educated men | 0.0231 | 0.043 | 0.0141 | -0.0388 | 0.000114 | 0.0135 | -0.000313 |
| Share of economically active women | 0.00216 | 0.00169** | -0.000252 | -0.00021 | 0.000878* | 0.0000692 | 0.0000614 |
| Kleibergen-Paap rk Wald F statistic | 2699.745 | 2699.745 | 2699.745 | 2699.745 | 2699.745 | 2699.745 | 2699.745 |
| Hansen J p-value | x | x | x | x | x | x | x |

*** 1% ** 5% * 10%. N=400, 20 years, 20 NUTS2 regions. Further controls include yearly dummies (partialled out). Standard errors are clustered at region level.

Table 9. IV: Full interaction model results for the UK (see Table 3 in Section 6.3). Interaction of exposure to robots with the initial share of workers employed out of manufacturing and interaction of exposure to robots with the initial ratio of women’s to men’s employment share in manufacturing.

| Covariate | TFR | FR 20-24 | FR 25-29 | FR 30-34 | FR 35-39 | FR 40-44 | FR 45+ |
| --- | --- | --- | --- | --- | --- | --- | --- |
| Exposure to robots | -0.0378*** | -0.0103 | -0.00187 | -0.00319 | -0.00486 | -0.00202 | -0.000238 |
| Exposure to robots # Initial share of workers out of manufacturing | 0.000420*** | 0.0000973 | 0.0000303 | 0.0000433 | 0.0000692 | 0.0000277 | 0.000000923 |
| Exposure to robots # Initial ratio of women's versus men's share in manufacturing | 0.0187* | 0.00487 | 0.000909 | 0.00303 | 0.00177 | 0.000732 | 0.000426** |
| Share of population aged 15-24 | 1.808 | -1.733*** | 0.66 | 2.552*** | 1.484 | 0.217 | -0.0622** |
| Share of population aged 25-49 | 0.0841 | -0.925 | -0.412 | 1.518* | 0.852 | 0.0115 | -0.0391 |
| Share of population aged 50+ | 2.541 | 0.172 | 0.395 | 1.541** | 0.964 | 0.0529 | -0.0334 |
| Share of highly educated population | -0.000252 | 0.000885 | -0.000423 | -0.000623 | -0.000839*** | -0.000135 | 0.0000313** |
| Ratio of share of highly-educated women to share of highly-educated men | 1.062*** | 0.221* | 0.244* | 0.367*** | 0.231** | 0.0468 | -0.00144 |
| Square of ratio of share of highly-educated women to share of highly-educated men | -0.506*** | -0.111** | -0.106 | -0.170*** | -0.113*** | -0.0239 | 0.000437 |
| Share of economically active women | -0.0021 | -0.00000855 | -0.000618 | -0.000567 | -0.000786** | -0.000247* | -0.0000168 |
| Kleibergen-Paap rk Wald F statistic | 140.698 | 140.698 | 140.698 | 140.698 | 140.698 | 140.698 | 140.698 |
| Hansen J p-value | 0.2151 | 0.1699 | 0.3893 | 0.0803 | 0.0995 | 0.3877 | 0.3232 |

*** 1% ** 5% * 10%. N=700, 20 years, 35 NUTS2 regions. Further controls include yearly dummies (partialled out). Standard errors are clustered at region level.

Table 10. IV: Full interaction model results for Poland and Czechia (see Table 3 in Section 6.3). Interaction of exposure to robots with the initial share of workers employed out of manufacturing and interaction of exposure to robots with the initial ratio of women’s to men’s employment share in manufacturing.

| Covariate | TFR | FR 20-24 | FR 25-29 | FR 30-34 | FR 35-39 | FR 40-44 | FR 45+ |
| --- | --- | --- | --- | --- | --- | --- | --- |
| Exposure to robots | 0.00195 | 0.000411 | -0.00436 | 0.00246 | 0.00178** | -0.000134 | -0.0000227 |
| Exposure to robots # Initial share of workers out of manufacturing | -0.0000722 | -0.0000313* | 0.0000517* | -0.0000380 | -0.0000319*** | 0.000000178 | -9.92e-08 |
| Exposure to robots # Initial ratio of women's versus men's share in manufacturing | 0.00402 | 0.00228*** | 0.000985 | 0.000298 | 0.000625 | 0.000137 | 0.0000230 |
| Share of population aged 15-24 | -2.668 | -1.465*** | 1.391* | 1.099 | -2.214*** | -0.761*** | -0.0916*** |
| Share of population aged 25-49 | -7.436*** | -3.153*** | -1.119 | 0.377 | -1.702*** | -0.644*** | -0.0838*** |
| Share of population aged 50+ | -4.702*** | -2.185*** | 0.299 | 0.523 | -1.787*** | -0.585*** | -0.0724*** |
| Share of highly educated population | 0.00316 | -0.000990 | 0.00109 | 0.00289** | 0.000517 | -0.0000873 | 0.0000279 |
| Ratio of share of highly-educated women to share of highly-educated men | -0.000703 | -0.0676 | -0.219*** | 0.125 | 0.122* | 0.0193 | 0.00444* |
| Square of ratio of share of highly-educated women to share of highly-educated men | 0.0120 | 0.0258* | 0.0880*** | -0.0430 | -0.0397* | -0.00782 | -0.00162* |
| Share of economically active women | 0.00732** | 0.00207** | 0.00532*** | 0.000436 | -0.000510 | 0.000108 | -0.0000199 |
| Kleibergen-Paap rk Wald F statistic | 3345.217 | 3345.217 | 3345.217 | 3345.217 | 3345.217 | 3345.217 | 3345.217 |
| Hansen J p-value | x | x | x | x | x | x | x |

*** 1% ** 5% * 10%. N=240, 10 years, 24 NUTS2 regions. Further controls include yearly dummies (partialled out). Standard errors are clustered at region level.

Table 11. IV: Full interaction model results for Germany (see Table 4 in Section 6.4). Interaction of exposure to robots with the share of highly educated population (ISCED 5-8).

| Covariate | TFR | FR 20-24 | FR 25-29 | FR 30-34 | FR 35-39 | FR 40-44 | FR 45+ |
| --- | --- | --- | --- | --- | --- | --- | --- |
| Exposure to robots | -0.00161*** | -0.000271* | -0.000111 | -0.000452** | -0.000435*** | -0.000142*** | -0.0000102** |
| Exposure to robots # Share of highly educated population | 0.0000538*** | 0.0000117** | 0.00000531 | 0.0000159** | 0.0000118*** | 0.00000339** | 0.000000334** |
| Share of population aged 15-24 | -1.009* | -0.792*** | -0.411*** | 0.750*** | -0.149** | -0.156*** | -0.0127*** |
| Share of population aged 25-49 | 1.616 | -0.182 | -0.622 | 1.368*** | 0.844*** | 0.0133 | -0.0190* |
| Share of population aged 50+ | 0.486 | -0.265** | -0.425** | 1.094*** | 0.127 | -0.170*** | -0.0227*** |
| Share of highly educated population | -0.00789*** | -0.00267*** | -0.00398*** | -0.00136** | 0.000484 | 0.000298 | 0.0000338 |
| Ratio of share of highly-educated women to share of highly-educated men | -0.555*** | -0.0571 | -0.131** | -0.195*** | -0.106*** | -0.0147 | -0.00203 |
| Square of ratio of share of highly-educated women to share of highly-educated men | 0.468*** | 0.0594** | 0.119*** | 0.145*** | 0.0861*** | 0.0148* | 0.00149* |
| Share of economically active women | 0.000785 | 0.000183 | 0.00142** | 0.0000187 | -0.000614 | -0.000357* | -0.0000443** |
| Kleibergen-Paap rk Wald F statistic | 903.478 | 903.478 | 903.478 | 903.478 | 903.478 | 903.478 | 903.478 |
| Hansen J p-value | 0.1312 | 0.3038 | 0.1785 | 0.2023 | 0.0447 | 0.1118 | 0.2687 |

*** 1% ** 5% * 10%. N=680, 20 years, 34 NUTS2 regions. Further controls include yearly dummies (partialled out). Standard errors are clustered at region level.

Table 12. IV: Full interaction model results for France (see Table 4 in Section 6.4). Interaction of exposure to robots with the share of highly educated population (ISCED 5-8).

| Covariate | TFR | FR 20-24 | FR 25-29 | FR 30-34 | FR 35-39 | FR 40-44 | FR 45+ |
| --- | --- | --- | --- | --- | --- | --- | --- |
| Exposure to robots | 0.00150** | 0.000579** | 0.00105*** | 0.000185 | -0.000274 | -0.000154** | -0.00000853 |
| Exposure to robots # Share of highly educated population | -0.0000536** | -0.0000248*** | -0.0000345** | -0.00000249 | 0.00000939 | 0.00000424* | 0.000000421 |
| Share of population aged 15-24 | -2.923 | -2.918*** | 0.148 | 0.315 | 0.275 | 0.00877 | 0.023 |
| Share of population aged 25-49 | -7.233*** | -3.825*** | -1.767** | -0.295 | -0.261 | -0.211 | 0.00000488 |
| Share of population aged 50+ | -6.211*** | -2.903*** | -1.032* | -0.576 | -0.654*** | -0.340*** | -0.0461*** |
| Share of highly educated population | 0.00403* | 0.00126* | 0.00174* | 0.00049 | 0.000319 | -0.0000491 | -0.00000386 |
| Ratio of share of highly-educated women to share of highly-educated men | 0.363 | 0.0322 | -0.0171 | 0.176* | 0.123** | 0.0536* | 0.00608 |
| Square of ratio of share of highly-educated women to share of highly-educated men | -0.171 | -0.0169 | 0.00187 | -0.0801* | -0.0532*** | -0.0227* | -0.00214 |
| Share of economically active women | -0.00171 | -0.000164 | 0.0000215 | -0.000909* | -0.000696*** | -0.000311*** | -0.000000551 |
| Kleibergen-Paap rk Wald F statistic | 1467.181 | 1467.181 | 1467.181 | 1467.181 | 1467.181 | 1467.181 | 1467.181 |
| Hansen J p-value | 0.2501 | 0.3655 | 0.1157 | 0.1607 | 0.2895 | 0.6744 | 0.4483 |

*** 1% ** 5% * 10%. N=440, 20 years, 22 NUTS2 regions. Further controls include yearly dummies (partialled out). Standard errors are clustered at region level.

Table 13. IV: Full interaction model results for Italy (see Table 4 in Section 6.4). Interaction of exposure to robots with the share of highly educated population (ISCED 5-8).

| Covariate | TFR | FR 20-24 | FR 25-29 | FR 30-34 | FR 35-39 | FR 40-44 | FR 45+ |
| --- | --- | --- | --- | --- | --- | --- | --- |
| Exposure to robots | -0.00292* | -0.00102 | -0.00124** | 0.000195 | -0.00016 | -0.000203* | 0.00000717 |
| Exposure to robots # Share of highly educated population | 0.000096 | 0.0000431* | 0.0000183 | -0.0000135 | 0.0000181 | 0.00000881** | -0.000000187 |
| Share of population aged 15-24 | -9.434*** | -4.280*** | -3.935*** | 0.367 | -0.752 | -0.487*** | -0.0325 |
| Share of population aged 25-49 | -7.489*** | -2.044*** | -4.449*** | -0.738** | 0.0488 | -0.211** | -0.0193 |
| Share of population aged 50+ | -6.478*** | -2.325*** | -3.034*** | -0.350** | -0.349** | -0.251*** | -0.0106 |
| Share of highly educated population | -0.00806* | -0.00284* | -0.00373** | -0.00106 | -0.00097 | 0.000602 | 0.000156 |
| Ratio of share of highly-educated women to share of highly-educated men | 0.00501 | -0.0872 | -0.0654 | 0.0993 | 0.0545 | -0.0212 | -0.00228 |
| Square of ratio of share of highly-educated women to share of highly-educated men | 0.0334 | 0.0423* | 0.0271 | -0.0285 | -0.00762 | 0.00939 | 0.000624 |
| Share of economically active women | 0.00324 | 0.00207*** | 0.000244 | -0.000195 | 0.0009 | 0.00012 | 0.0000604 |
| Kleibergen-Paap rk Wald F statistic | 393.028 | 393.028 | 393.028 | 393.028 | 393.028 | 393.028 | 393.028 |
| Hansen J p-value | 0.4075 | 0.2984 | 0.6674 | 0.3396 | 0.1435 | 0.3712 | 0.3528 |

*** 1% ** 5% * 10%. N=400, 20 years, 20 NUTS2 regions. Further controls include yearly dummies (partialled out). Standard errors are clustered at region level.

Table 14. IV: Full interaction model results for the UK (see Table 4 in Section 6.4). Interaction of exposure to robots with the share of highly educated population (ISCED 5-8).

| Covariate | TFR | FR 20-24 | FR 25-29 | FR 30-34 | FR 35-39 | FR 40-44 | FR 45+ |
| --- | --- | --- | --- | --- | --- | --- | --- |
| Exposure to robots | 0.000259 | -0.000494 | 0.00171* | 0.000626 | 0.0000798 | -0.00016 | -0.0000943** |
| Exposure to robots # Share of highly educated population | 0.0000315 | -0.00000892 | -0.000023 | 0.0000146 | 0.0000236 | 0.0000124 | 0.00000205*** |
| Share of population aged 15-24 | 0.646 | -2.084*** | 0.553 | 2.414*** | 1.408 | 0.193 | -0.0781*** |
| Share of population aged 25-49 | -0.342 | -1.124* | -0.525 | 1.498* | 0.906 | 0.0454 | -0.0419 |
| Share of population aged 50+ | 2.121 | 0.0141 | 0.333 | 1.505** | 0.97 | 0.0612 | -0.0378 |
| Share of highly educated population | -0.000237 | 0.00111 | -0.0000925 | -0.000785 | -0.00108** | -0.000271** | 0.00000235 |
| Ratio of share of highly-educated women to share of highly-educated men | 1.026*** | 0.216* | 0.251* | 0.365*** | 0.217** | 0.0404 | -0.000719 |
| Square of ratio of share of highly-educated women to share of highly-educated men | -0.486*** | -0.109* | -0.11 | -0.168*** | -0.106** | -0.0205 | 0.000192 |
| Share of economically active women | -0.00254 | -0.000141 | -0.000703 | -0.000599 | -0.000816** | -0.000253* | -0.0000166 |
| Kleibergen-Paap rk Wald F statistic | 106.778 | 106.778 | 106.778 | 106.778 | 106.778 | 106.778 | 106.778 |
| Hansen J p-value | 0.0832 | 0.3896 | 0.1509 | 0.1971 | 0.1096 | 0.0760 | 0.2256 |

*** 1% ** 5% * 10%. N=700, 20 years, 35 NUTS2 regions. Further controls include yearly dummies (partialled out). Standard errors are clustered at region level.

Table 15. IV: Full interaction model results for Poland and Czechia (see Table 4 in Section 6.4). Interaction of exposure to robots with the share of highly educated population (ISCED 5-8).

| Covariate | TFR | FR 20-24 | FR 25-29 | FR 30-34 | FR 35-39 | FR 40-44 | FR 45+ |
| --- | --- | --- | --- | --- | --- | --- | --- |
| Exposure to robots | -0.000180 | 0.000386 | -0.00182*** | 0.000231 | 0.000660*** | 0.0000187 | -0.0000343*** |
| Exposure to robots # Share of highly educated population | 0.0000213 | -0.0000195 | 0.0000727** | 0.00000502 | -0.0000241** | -0.00000291 | 0.00000119** |
| Share of population aged 15-24 | -1.279 | -1.324* | 1.526** | 1.855* | -2.075*** | -0.825*** | -0.0714*** |
| Share of population aged 25-49 | -6.913*** | -3.142*** | -1.102 | 0.726 | -1.663*** | -0.681*** | -0.0746*** |
| Share of population aged 50+ | -3.418** | -2.027*** | 0.425 | 1.195 | -1.650*** | -0.638*** | -0.0544*** |
| Share of highly educated population | 0.00276 | -0.00102 | 0.000252 | 0.00297*** | 0.000713* | -0.0000897 | 0.0000178 |
| Ratio of share of highly-educated women to share of highly-educated men | 0.188 | -0.00249 | -0.190*** | 0.180 | 0.150** | 0.0194 | 0.00613** |
| Square of ratio of share of highly-educated women to share of highly-educated men | -0.0585 | 0.00129 | 0.0770*** | -0.0635 | -0.0502* | -0.00787 | -0.00225** |
| Share of economically active women | 0.00684** | 0.00196* | 0.00479*** | 0.000406 | -0.000420 | 0.000109 | -0.0000286 |
| Kleibergen-Paap rk Wald F statistic | 204.214 | 204.214 | 204.214 | 204.214 | 204.214 | 204.214 | 204.214 |
| Hansen J p-value | 0.3575 | 0.3414 | 0.3279 | 0.5557 | 0.3803 | 0.3581 | 0.2856 |

*** 1% ** 5% * 10%. N=240, 10 years, 24 NUTS2 regions. Further controls include yearly dummies (partialled out). Standard errors are clustered at region level.

Table 16. IV: Full interaction model results for Germany (see Table 5 in Section 6.5). Interaction of exposure to robots with the share of workers employed in technology and knowledge-intensive sectors.

| Covariate | TFR | FR 20-24 | FR 25-29 | FR 30-34 | FR 35-39 | FR 40-44 | FR 45+ |
| --- | --- | --- | --- | --- | --- | --- | --- |
| Exposure to robots | -0.0000577 | 0.0000956 | 0.000152** | -0.0000296 | -0.000149*** | -0.0000528*** | -0.00000342* |
| Exposure to robots # Share of workers employed in technology- and knowledge-intensive sectors | -0.0000186 | -0.0000136 | -0.0000498*** | 0.00000508 | 0.0000223* | 0.00000411 | 0.00000104** |
| Share of population aged 15-24 | -1.140** | -0.826*** | -0.540*** | 0.713*** | -0.106 | -0.147*** | -0.0105** |
| Share of population aged 25-49 | 2.088* | -0.144 | -0.784* | 1.582*** | 1.055*** | 0.0626 | -0.0118 |
| Share of population aged 50+ | 0.482 | -0.301* | -0.607*** | 1.130*** | 0.229* | -0.149*** | -0.0186*** |
| Share of highly educated population | -0.00175 | -0.00129** | -0.00282*** | 0.000422 | 0.00149*** | 0.000607*** | 0.0000585*** |
| Ratio of share of highly-educated women to share of highly-educated men | -0.661*** | -0.0830** | -0.149** | -0.222*** | -0.126*** | -0.0211 | -0.00251* |
| Square of ratio of share of highly-educated women to share of highly-educated men | 0.523*** | 0.0720*** | 0.128*** | 0.161*** | 0.0958*** | 0.0178** | 0.00172** |
| Share of economically active women | -0.000677 | -0.000156 | 0.000951 | -0.000406 | -0.00073 | -0.000402** | -0.0000456** |
| Share of workers employed in technology- and knowledge-intensive sectors | 0.00563 | 0.00352 | 0.00432** | -0.00126 | -0.0000482 | 0.000293 | -0.0000361 |
| Kleibergen-Paap rk Wald F statistic | 414.164 | 414.164 | 414.164 | 414.164 | 414.164 | 414.164 | 414.164 |
| Hansen J p-value | 0.2354 | 0.5993 | 0.0961 | 0.1332 | 0.1012 | 0.1885 | 0.0672 |

*** 1% ** 5% * 10%. N=680, 20 years, 34 NUTS2 regions. Further controls include yearly dummies (partialled out). Standard errors are clustered at region level.

Table 17. IV: Full interaction model results for France (see Table 5 in Section 6.5). Interaction of exposure to robots with the share of workers employed in technology and knowledge-intensive sectors.

| Covariate | TFR | FR 20-24 | FR 25-29 | FR 30-34 | FR 35-39 | FR 40-44 | FR 45+ |
| --- | --- | --- | --- | --- | --- | --- | --- |
| Exposure to robots | -0.000147 | -0.000187* | 0.0000601 | 0.00013 | -0.0000432 | -0.0000720*** | -0.00000177 |
| Exposure to robots # Share of workers employed in technology- and knowledge-intensive sectors | 0.0000721 | 0.0000333 | 0.0000178 | -1.27E-06 | 0.0000114 | 0.0000115* | 0.00000163 |
| Share of population aged 15-24 | -3.397 | -3.083*** | -0.134 | 0.265 | 0.328 | 0.0253 | 0.0271 |
| Share of population aged 25-49 | -7.336*** | -3.849*** | -1.911** | -0.326 | -0.182 | -0.158 | 0.00782 |
| Share of population aged 50+ | -6.092*** | -2.814*** | -1.039* | -0.607 | -0.611*** | -0.300*** | -0.0389*** |
| Share of highly educated population | 0.00179 | 0.000256 | 0.000316 | 0.000368 | 0.000690* | 0.000118 | 0.0000139 |
| Ratio of share of highly-educated women to share of highly-educated men | 0.225 | -0.0403 | -0.103 | 0.175* | 0.147*** | 0.0642** | 0.00663* |
| Square of ratio of share of highly-educated women to share of highly-educated men | -0.109 | 0.0149 | 0.04 | -0.0791* | -0.0634*** | -0.0273** | -0.00240* |
| Share of economically active women | -0.00103 | 0.000161 | 0.000415 | -0.000883* | -0.000779*** | -0.000344*** | -0.00000262 |
| Share of workers employed in technology- and knowledge-intensive sectors | -0.00207 | -0.00183 | -0.000152 | 0.000656 | -0.000469 | -0.000504 | -0.000116 |
| Kleibergen-Paap rk Wald F statistic | 1620.387 | 1620.387 | 1620.387 | 1620.387 | 1620.387 | 1620.387 | 1620.387 |
| Hansen J p-value | 0.4884 | 0.4575 | 0.4411 | 0.6228 | 0.5744 | 0.5741 | 0.5064 |

*** 1% ** 5% * 10%. N=440, 20 years, 22 NUTS2 regions. Further controls include yearly dummies (partialled out). Standard errors are clustered at region level.

Table 18. IV: Full interaction model results for Italy (see Table 5 in Section 6.5). Interaction of exposure to robots with the share of workers employed in technology and knowledge-intensive sectors.

| Covariate | TFR | FR 20-24 | FR 25-29 | FR 30-34 | FR 35-39 | FR 40-44 | FR 45+ |
| --- | --- | --- | --- | --- | --- | --- | --- |
| Exposure to robots | -0.00116* | -0.000131 | -0.00117*** | -0.000172 | 0.000373*** | -0.00000882 | 0.00000238 |
| Exposure to robots # Share of workers employed in technology- and knowledge-intensive sectors | 0.00000518 | -0.0000191 | 0.0000995 | 0.0000193 | -0.0000753 | -0.0000135 | 0.00000231 |
| Share of population aged 15-24 | -9.110*** | -4.234*** | -3.602*** | 0.452 | -0.918** | -0.497*** | -0.0342 |
| Share of population aged 25-49 | -6.932*** | -1.848*** | -4.149*** | -0.747** | -0.0103 | -0.190** | -0.02 |
| Share of population aged 50+ | -6.601*** | -2.414*** | -2.950*** | -0.291 | -0.460*** | -0.278*** | -0.00995 |
| Share of highly educated population | -0.00172 | -0.000135 | -0.00237** | -0.00171* | 0.0000626 | 0.00116*** | 0.000128* |
| Ratio of share of highly-educated women to share of highly-educated men | -0.0485 | -0.105 | -0.0995 | 0.0994 | 0.0637 | -0.0226 | -0.00246 |
| Square of ratio of share of highly-educated women to share of highly-educated men | 0.054 | 0.0491* | 0.0405 | -0.0288 | -0.0112 | 0.00994 | 0.000766 |
| Share of economically active women | 0.0033 | 0.00199** | 0.000503 | -0.0000225 | 0.000648 | 0.0000738 | 0.000046 |
| Share of workers employed in technology- and knowledge-intensive sectors | -0.00621 | 0.000756 | -0.0111** | -0.00445 | 0.00792** | 0.00111 | 0.000212 |
| Kleibergen-Paap rk Wald F statistic | 1518.104 | 1518.104 | 1518.104 | 1518.104 | 1518.104 | 1518.104 | 1518.104 |
| Hansen J p-value | 0.4369 | 0.4616 | 0.3405 | 0.5515 | 0.2274 | 0.3733 | 0.2997 |

*** 1% ** 5% * 10%. N=400, 20 years, 20 NUTS2 regions. Further controls include yearly dummies (partialled out). Standard errors are clustered at region level.

Table 19. IV: Full interaction model results for the UK (see Table 5 in Section 6.5). Interaction of exposure to robots with the share of workers employed in technology and knowledge-intensive sectors.

| Covariate | TFR | FR 20-24 | FR 25-29 | FR 30-34 | FR 35-39 | FR 40-44 | FR 45+ |
| --- | --- | --- | --- | --- | --- | --- | --- |
| Exposure to robots | 0.00161 | -0.000795 | 0.00122 | 0.00151 | 0.000712 | 0.000163 | -0.00000639 |
| Exposure to robots # Share of workers employed in technology- and knowledge-intensive sectors | 0.00000614 | 0.00000432 | -0.000202* | -0.0000451 | 0.000123 | 0.0000709* | -6.81E-08 |
| Share of population aged 15-24 | 0.495 | -2.144*** | 0.293 | 2.333** | 1.564* | 0.27 | -0.0831*** |
| Share of population aged 25-49 | -0.679 | -1.280* | -0.924 | 1.439 | 1.139 | 0.146 | -0.0507* |
| Share of population aged 50+ | 1.941 | -0.0794 | 0.119 | 1.468** | 1.102 | 0.118 | -0.0422* |
| Share of highly educated population | 0.0000447 | 0.000878 | -0.000413 | -0.000479 | -0.000754** | -0.000123 | 0.0000299 |
| Ratio of share of highly-educated women to share of highly-educated men | 1.003*** | 0.182 | 0.186 | 0.373*** | 0.264*** | 0.0614* | -0.000105 |
| Square of ratio of share of highly-educated women to share of highly-educated men | -0.474*** | -0.0908* | -0.0786 | -0.173*** | -0.129*** | -0.0306** | -0.000151 |
| Share of economically active women | -0.00270* | -0.000211 | -0.000952* | -0.00061 | -0.000678* | -0.00019 | -0.0000206 |
| Share of workers employed in technology- and knowledge-intensive sectors | 0.00376 | 0.00340* | 0.00706*** | -0.00106 | -0.00465** | -0.00199*** | -0.00000187 |
| Kleibergen-Paap rk Wald F statistic | 113.586 | 113.586 | 113.586 | 113.586 | 113.586 | 113.586 | 113.586 |
| Hansen J p-value | 0.0949 | 0.2479 | 0.3943 | 0.1157 | 0.2888 | 0.3732 | 0.1786 |

*** 1% ** 5% * 10%. N=700, 20 years, 35 NUTS2 regions. Further controls include yearly dummies (partialled out). Standard errors are clustered at region level.

Table 20. IV: Full interaction model results for Poland and Czechia (see Table 5 in Section 6.5). Interaction of exposure to robots with the share of workers employed in technology and knowledge-intensive sectors.

| Covariate | TFR | FR 20-24 | FR 25-29 | FR 30-34 | FR 35-39 | FR 40-44 | FR 45+ |
| --- | --- | --- | --- | --- | --- | --- | --- |
| Exposure to robots | 0.00119 | 0.000252 | -0.000470 | 0.000960 | 0.000393* | -0.0000861** | -0.0000300*** |
| Exposure to robots # Share of workers employed in technology- and knowledge-intensive sectors | -0.000313 | -0.0000393 | -0.0000320 | -0.000215 | -0.0000573 | 0.0000171 | 0.00000623** |
| Share of population aged 15-24 | -2.890 | -1.026 | 0.0226 | 0.862 | -1.920*** | -0.683*** | -0.0503 |
| Share of population aged 25-49 | -8.137*** | -2.969*** | -1.901 | -0.0993 | -1.689*** | -0.589*** | -0.0536* |
| Share of population aged 50+ | -4.727** | -1.767*** | -0.889 | 0.403 | -1.491*** | -0.517*** | -0.0369 |
| Share of highly educated population | 0.00188 | -0.00116 | 0.000722 | 0.00223** | 0.000320 | -0.0000818 | 0.0000352 |
| Ratio of share of highly-educated women to share of highly-educated men | 0.167 | 0.0100 | -0.261*** | 0.177 | 0.174*** | 0.0232 | 0.00539** |
| Square of ratio of share of highly-educated women to share of highly-educated men | -0.0521 | -0.00350 | 0.103*** | -0.0635 | -0.0594** | -0.00917* | -0.00193** |
| Share of economically active women | 0.00730** | 0.00189* | 0.00523*** | 0.000670 | -0.000488 | 0.0000739 | -0.0000298 |
| Share of workers employed in technology- and knowledge-intensive sectors | 0.0161* | 0.00161 | -0.00128 | 0.0117*** | 0.00355*** | -0.000282 | -0.0000526 |
| Kleibergen-Paap rk Wald F statistic | 94.567 | 94.567 | 94.567 | 94.567 | 94.567 | 94.567 | 94.567 |
| Hansen J p-value | 0.2895 | 0.3902 | 0.2151 | 0.4784 | 0.3056 | 0.6450 | 0.4738 |

*** 1% ** 5% * 10%. N=240, 10 years, 24 NUTS2 regions. Further controls include yearly dummies (partialled out). Standard errors are clustered at region level.

*please add the models from the Excel file here: tables 21-45*
